# The First Molecular Detection of *Aedes albopictus* in Sudan Associates with Increased Outbreaks of Chikungunya and Dengue

**DOI:** 10.3390/ijms231911802

**Published:** 2022-10-05

**Authors:** Ayman Ahmed, Mustafa Abubakr, Hamza Sami, Isam Mahdi, Nouh S. Mohamed, Jakob Zinsstag

**Affiliations:** 1 Institute of Endemic Diseases, University of Khartoum, Khartoum 11111, Sudan; 2 Swiss Tropical and Public Health Institute (Swiss TPH), CH-4123 Allschwil, Switzerland; 3 Faculty of Science, University of Basel, Petersplatz 1, CH-4001 Basel, Switzerland; 4 Molecular Biology Unit, Sirius Training and Research Centre, Khartoum 11111, Sudan; 5 Directorate of Environmental Health, Federal Ministry of Health, Khartoum 11111, Sudan; 6 Directorate of the Integrated Vector Management (IVM), Federal Ministry of Health, Khartoum 11111, Sudan

**Keywords:** invasive diseases vectors, *Aedes aegypti*, *Aedes vexans*, *Aedes vittatus*, *Aedes africanus*, *Aedes metalicus*, *Aedes luteocephalus*, *Anopheles stephensi*, arboviruses, haplotype analysis, phylogenetic analysis, One Health, Sudan

## Abstract

As part of our surveys of the invasive malaria vector *Anopheles stephensi* in four Sudanese states, including North and South Kordofan, Sennar, and White Nile, we collected 166 larvae. Our morphological identification confirmed that 30% of the collected mosquito samples were *Anopheles* species, namely *An. gambiae s.l.* and *An. stephensi*, while the 117 *Aedes* specimens were *Ae. luteocephalus* (39%), *Ae. aegypti* (32%), *Ae. vexans* (9%), *Ae. vittatus* (9%), *Ae. africanus* (6%), *Ae. metalicus* (3%), and *Ae. albopictus* (3%). Considering the serious threat of *Ae. albopictus* emergence for the public health in the area and our limited resources, we prioritized *Ae. albopictus* samples for further genomic analysis. We extracted the DNA from the three specimens and subsequently sequenced the cytochrome oxidase 1 (CO1) gene and confirmed their identity as *Aedes albopictus* and their potential origin by phylogenetic and haplotype analyses. *Aedes albopictus*, originating from Southeast Asia, is an invasive key vector of chikungunya and dengue. This is the first report and molecular characterization of *Ae. albopictus* from Sudan. Our sequences cluster with populations from the Central African Republic and La Réunion. Worryingly, this finding associates with a major increase in chikungunya and dengue outbreaks in rural areas of the study region and might be linked to the mosquito’s spread across the region. The emergence of *Ae. albopictus* in Sudan is of serious public health concern and urges for the improvement of the vector surveillance and control system through the implementation of an integrated molecular xenosurveillance. The threat of major arboviral diseases in the region underlines the need for the institutionalization of the One Health strategy for the prevention and control of future pandemics.

## 1. Introduction

The global disease burden and distribution of arthropod-borne viruses (arboviruses) is rapidly growing. This growth is driven primarily by the spread of the two key invasive disease vectors, *Aedes aegypti* and *Ae. albopictus,* and by the spread of new and re-emerging viruses through international travel [1]. Arboviruses are infecting a wide range of hosts, including humans and other vertebrates, and arthropod disease vectors [2]. Some of the arboviruses are posing a serious global health threat, such as, chikungunya (CHIKV), Crimean–Congo hemorrhagic fever (CCHF), dengue (DENV), yellow fever (YFV), and Zika (ZIKV) virus infections [3,4]. Other arboviruses, such as Rift Valley fever (RVF), African swine fever, bluetongue, as well as Marburg (MBGV) and Schmallenberg viruses, are having devastating economic impacts because of their high morbidity and mortality among domestic animals, including cattle, sheep, and goats [5,6,7]. While some arboviruses, such as the Shuni virus, Wesselsbron virus, and West Nile virus (WNV), severely affect domestic and wildlife animals [8,9].

*Aedes albopictus* is a more recent species to Africa that only emerged in the area during the recent three decades [1,10]. *Aedes albopictus* is deemed to be the most invasive insect species and it is estimated that it will be present in 197 countries by 2080 [1]. Interestingly, the presence, spread, and abundance of these invasive disease vectors are mostly associated with socioeconomically disadvantaged communities [11,12]. In Europe, where *Ae. albopictus* is already widely spread, surveillance and control programmes were designed and implemented to slow down its spread and minimise the public health risk of diseases transmission [13,14,15,16]. Unfortunately, such a programme does not yet exist in low and middle-income countries due to resources limitations and the fact that health systems are heavily burdened by malaria, particularly in Africa [17]. Therefore, invasive vectors of diseases are spreading undetected until they are established locally and disease outbreaks occur [18,19,20].

During recent years, the distribution and burden of arboviruses in Sudan increased remarkably, with several annual outbreaks reported from across the country [21]. The emerging and re-emerging arboviral diseases in Sudan include chikungunya, CCHF, dengue, RVF, and WNV [4,5,17,22,23,24,25,26]. However, the surveillance system of arboviral disease vectors in the country is outdated, with only one comprehensive survey of the species vector composition and distribution of *Aedes* mosquitoes dating back to 1955 [27].

As part of our surveys of the invasive malaria vector *Anopheles stephensi*, we also identified, for the first time, the presence of *Ae. albopictus* in Sudan. Additionally, we confirmed the presence of other *Aedes* arbovirus vectors.

## 2. Results

**Figure 1:**
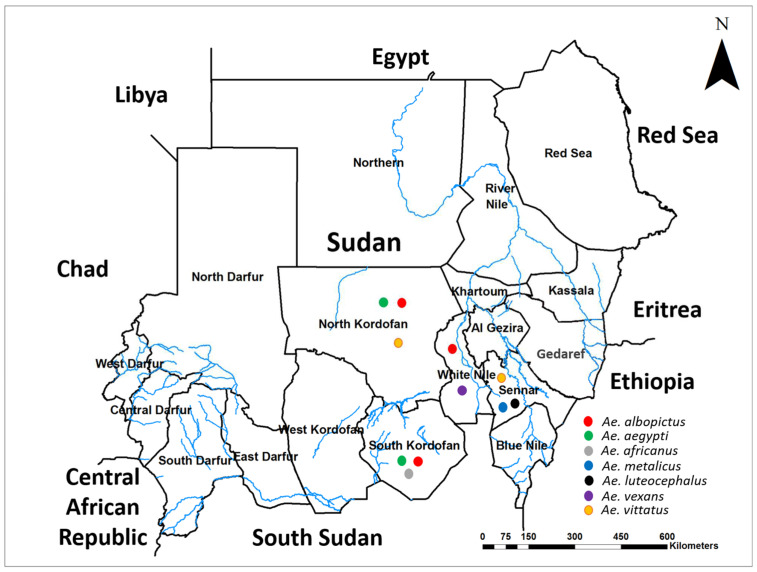
Map of Sudan showing the geographical distribution of identified *Aedes* mosquitoes in the study area.

We collected 166 larvae from human-made water containers in four Sudanese states, including North and South Kordofan, Sennar, and White Nile between 3 October and 29 December 2021. Thirty percent were *Anopheles* and 70% were *Aedes* mosquitoes. We morphologically identified the *Anopheles* species as *An. gambiae* s.l. and *An. stephensi*, while the 117 *Aedes* species were *Ae. luteocephalus* (39%), *Ae. aegypti* (32%), *Ae. vexans* (9%), *Ae. vittatus* (9%), *Ae. africanus* (6%), *Ae. metalicus* (3%), and—more importantly—3% were identified as *Ae. albopictus*. Figure 1 shows their geographical distribution per the states.

Considering its public health significance and our limited resources, following the morphological identification of emerged adult mosquitoes, we prioritised specimens that morphologically identified as *Ae. albopictus* for further sequence analysis. We sequenced an about 450 bp long PCR amplicon of the mitochondrial cytochrome oxidase 1 (CO1) gene and searched for similar sequences in the GenBank database at NCBI (https://blast.ncbi.nlm.nih.gov/nucleotide, accessed on 13 April 2022) using BLAST [28]. This confirmed the identity of the three morphologically identified *Ae. albopictus* samples, and we deposited the sequences in NCBI (accession numbers: ON248551-ON248553).

### 2.1. Phylogenetic Analysis

**Figure 2:**
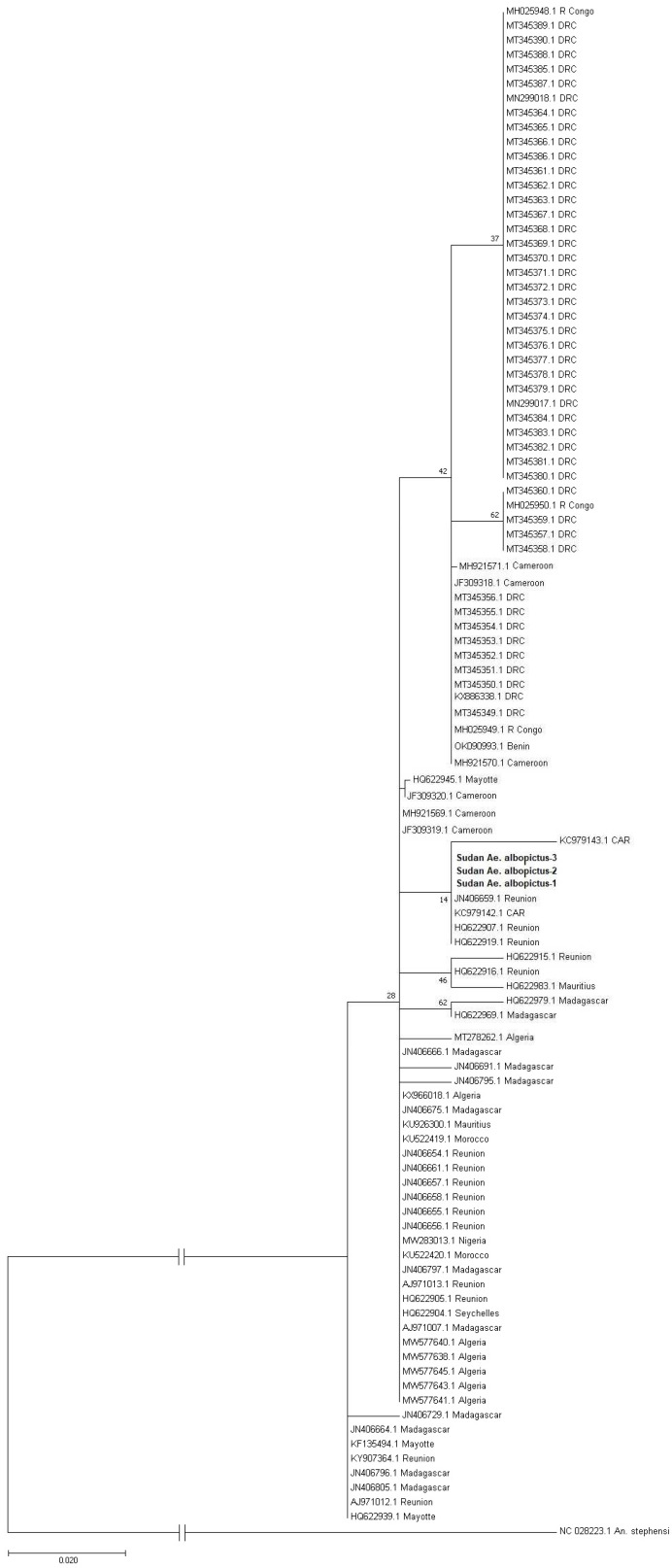
Maximum likelihood phylogenetic tree showing the relation between the Sudanese *Ae. albopictus* sequences and 103 African reference sequences. The Sudan *Ae. albopictus* sequences are highlighted in bold. The reference sequences along with their accession numbers and origin of the isolate are shown for each. DRC: Demogratic Republic of Congo, CAR: Central African Republic, R Congo: Republic of Congo. *An. stephensi* (Accession No. NC_028223.1) was used as an outgroup taxon. The branch to the outgroup was shortened by 0.020 substitutions per site. The bootstrap consensus tree inferred from 1000 replicates.

We constructed a phylogenetic tree and found that our three sequences are mainly clustered with sequences originating from the Central African Republic and La Réunion (Figure 2).

### 2.2. Haplotype Analysis and Global Network

**Figure 3:**
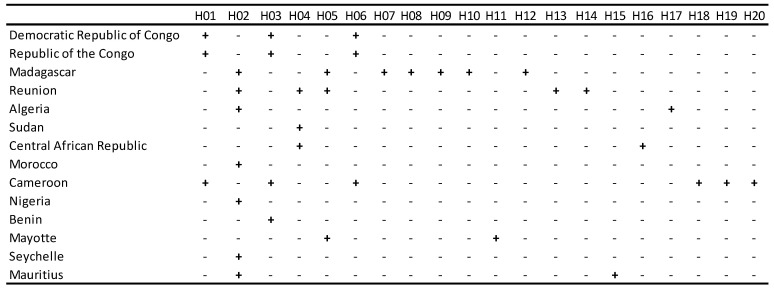
*Aedes albopictus* haplotype distribution among African countries. Countries or regions where a haplotype is present are indicated by (+), whereas, the absence of the haplotype is indicated by (−).

The results of the haplotype numbers and haplotype diversity show the presence of 20 haplotypes among *Ae. albopictus* in Africa with a haplotype diversity of 0.83 ± 0.02 (mean ± sd), indicating a low diversity between the haplotypes. Tajima’s D is −2.64, while Fu and Li’s D statistic is −8.66 and Fu and Li’s F statistic is −7.35. All tests were statistically significant, with the *p* value < 0.05. The distribution of haplotypes according to each country showed that H02 was reported from seven regions, followed by H03 in four regions (Figure 3).

According to the haplotype diversity (Hd), Hd was high in Algeria and low in other counties, such as Madagascar, Mayotte, and La Réunion. Additionally, several counties presented very low haplotype diversity, such as Cameroon, the Central African Republic, Mauritius, and the Republic of the Congo. Although Sudan is presented with three sequences, all three sequences are one haplotype. Evolutionary analysis using Tajima D and Fu’s statistics suggests different scenarios of a population bottleneck event and population expansion in the Democratic Republic of the Congo. The evolutionary analysis was not applicable for Sudan mainly because of the limited sample size; the three sequences constituted one haplotype (Table 1). However, analyzing the haplotypes of *Ae. albopictus* in other African countries indicates that *Ae. albopictus* populations are having a wide diversity and evolutionary dynamic.

### 2.3. Population Diversity and Evolutionary Characteristics

Further investigation of the population diversity showed that the calculated Fst values confirm the wide genetic variation among the African *Ae. albopictus* populations (Table 2).

**Figure 4:**
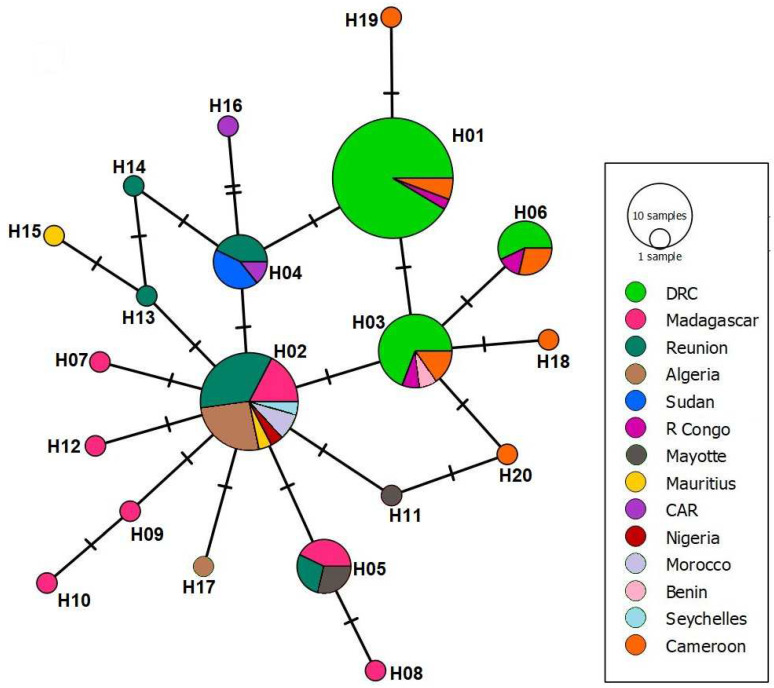
Parsimony haplotype network of the 103 African *Ae. albopictus* reference sequences and the 3 Sudanese *Ae. albopictus* sequences. Haplotypes of each region are presented using color coding. The number of each haplotype is written next to its representing node. Hatch marks in lines linking between the haplotypes are indicative of the numbers of nucleotide diversity. DRC: Democratic Republic of the Congo, R Congo: Republic of the Congo, and CAR: Central African Republic.

The constructed haplotypes network reveals the expansion of H02 in a star-like network, suggesting that H02 is the main haplotype from which the other haplotypes evolutionary expanded (Figure 4).

### 2.4. Recent Increase in the Burden and Spread of CHIKV in Sudan and the Neighboring Countries

**Figure 5:**
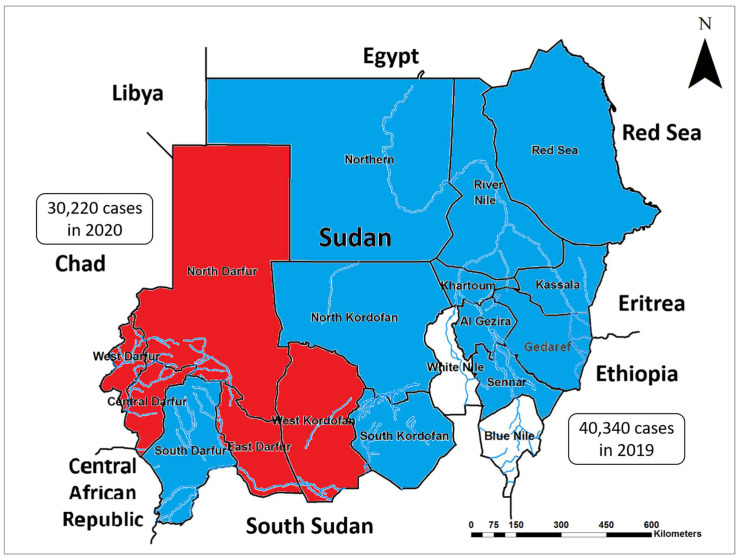
Map of Sudan showing the geographical distribution of chikungunya virus infections before (blue) and after (red) 2020 throughout the country. Up to now, no transmission is reported in the two states without color: White Nile and Blue Nile states. The number of cases reported during two outbreaks in the neighboring countries, Chad and Ethiopia, are included in the textboxes.

Reviewing the national outbreak reports, the literature and online records indicate that before 2020, no case of CHIKV was reported from any states in West Sudan (i.e., Central, East, North, and West Darfur and West Kordofan) [21]. However, between 2020 and 2022, outbreaks of CHIKV were reported in these states (Figure 5). This underscores the rapid expansion of the geographical distribution of this virus in the country. Additionally, we identified several outbreaks of CHIKV that occurred during the recent two years in the region. These outbreaks include 45 cases in Kenya in 2018; 48,734 cases in Sudan in 2018–2019; 40,340 and 11,230 cases in Ethiopia and the Republic of Congo in 2019, respectively, and 30,220 cases in Chad in 2020.

## 3. Discussion

This study offers the first molecular confirmation about the presence of the invasive arbovirus vector *Ae. albopictus* in Sudan. However, there were epidemiological indicators about the spread of this invasive vector in the area. Some of these indicators are regional, such as the recent substantial growth of chikungunya outbreaks in East Africa, including Chad, Ethiopia, Kenya, Republic of Congo, and Sudan [17,29,30,31,32]. Additionally, local indicators include the geographical expansion of arboviral disease distributions in the country indicated by the emergence and re-emergence of several *Aedes*-borne arboviral diseases and development of outbreaks in rural areas throughout the country [5,17,21,22,23,24,25]. This is further underscored by the emergence of chikungunya and dengue fever outbreaks in several states in Western Sudan, including the study area for the first time during the last five years [5,17,21,22,23,24,25,33].

The role of the other species of *Aedes* mosquitoes, the presence of which was confirmed in this study, in the outbreaks of arboviral diseases that recently developed throughout the country should not be ignored because they are known vectors of several arboviruses. For instance, *Ae. aegypti* is an endemic vector in Africa that transmits major human viruses, such as CHIKV, DENV, YFV, and ZIKV [21]. However, it is a globally known invasive vector for important viruses including CHIKV, DENV, MBGV, RVF, and WNV, African horse sickness virus (AHSV), Epizootic hemorrhagic disease virus (EHDV), and Mayaro virus (MAYV), as well as Eastern Equine Encephalitis virus (EEEV), Japanese Encephalitis virus (JBEV), Murray Valley Encephalitis virus (MVEV), and Venezuelan Equine Encephalitis virus (VEEV) [34]. Interestingly, *Ae. africanus* is an African native vector of arboviruses that is also associated with the transmission of CHIKV, DENV, RVF, YFV, and ZIKV [35]. Furthermore, *Ae. luteocephalus* is involved in the transmission of chikungunya, dengue, yellow fever, and Zika viruses [36]. Moreover, *Ae. vittatus* is another African vector of arboviruses that is mainly involved in the transmission of CHIKV, DENV, JBEV, WNV, YFV, and ZIKV [37]. In addition to transmitting the EEEV, JBEV, St. Louis Encephalitis virus (SLEV), VEEV, WEEV, WNV, and ZIKV [38], while *Ae. vexans* is known as the initial vector for RVF outbreaks because RVF virus can be maintained vital in the mosquitoes eggs that can withstand desiccation in dry land for years [39]. This is the reason behind the inter-epizootic and inter-epidemic periods between RVF outbreaks, because *Ae. vexans* act as an alternative host [39,40,41]. However, a main limitation of this study is that we did not have enough resources to collect the adult mosquitoes of these confirmed species of *Aedes* vectors to incriminate them and investigate their role in the local transmission of arboviruses. Unfortunately, the current surveillance and control systems for human and animal diseases, as well as their vectors in the country, are separate and inadequate, limiting their ability to detect and report diseases in a timely manner in order to take effective control measures [21,42,43]. Furthermore, in addition to the heavy burden of malaria that overwhelmingly occupies the healthcare system, the country suffers from armed conflict and climate change, as evidenced by the recent frequency of extreme weather events across the country [33,44,45]. These factors together favor the spread and increased abundance of disease vectors, particularly the invasive vectors, such as *Ae. albopictus* and *An. stephensi* [46,47,48,49,50]. Nonetheless, the delay in detecting the emergence of these vectors of arboviral diseases that we report here could be attributed to the partial freeze in environmental and public health services due to the global COVID-19 pandemic [51]. Therefore, the need for a One Health-integrated surveillance and response system for diseases and their vectors is extreme and urgent [50,52,53,54].

The phylogenetic analyses show that the Sudanese specimens of *Ae. albopictus* closely cluster with sequences from La Réunion and the Central African Republic. Considering the open borders and high dynamics of human and animal populations between the Central African Republic and Sudan, and that all the current three samples were collected relatively close to these borders, it is likely that it was introduced into Sudan from the Central African Republic. In addition, the western borders of Sudan had suffered from an armed-conflict that persisted since 2003, and several diseases and disease vectors emerged in the area. The emergence and spread of several zoonotic diseases, such as CCHF, dengue, hepatitis E virus, RVF, YF, and WNV in Sudan, were associated with the environmental and socioeconomic changes due to the war-induced humanitarian crisis [4,5,21,22,23,24,25,33,55,56,57]. Climate change is also playing a major role in the spread of infectious diseases and their vectors in the country and globally [17,33,58,59,60]. Our haplotype analysis identified a single haplotype in the country, mainly because of the small sample size; therefore, we cannot exclude the presence of other haplotypes in the country, particularly since at least around 20 haplotypes exist among the African populations of *Ae. albopictus* (Figure 4) [61,62,63,64,65,66]. Our haplotype network analysis suggested that H02 is the central haplotype from which other haplotypes were driven. Interestingly, this is further supported by the fact that H02 is exclusively reported from islands and coastal countries where the initial introduction of this invasive vector is highly likely (Figure 4).

Unlike *Ae. aegypti*, the other identified species of *Aedes* mosquitoes are zoophilic, which means they prefer to feed on a non-human host, yet readily feed on humans according to host availability, making them of high risk for the transmission of zoonoses. Particularly, nearly a third of the 545 suspected arboviruses are zoonotic in nature, readily infecting humans, domestic, and wildlife animals [3]. Some of these arboviruses have a wide range of hosts and vectors, making them challenging to prevent and control [2]. However, due to their evolution, high rate of mutations, and capacity to spillover and spill back, the host range and competency of different arthropods to transmit the different arboviruses is persistently expanding [67,68]. Considering the climate change, rapid unplanned urbanization, globalization, conflicts, and increased size of population lives in humanitarian crisis with limited services, now more than ever, there is a severe need for a countrywide One Health-integrated surveillance and response system to prevent future pandemics [52,54,69]. Particularly, the geographical location of Sudan, its size spanning over different ecological zones, including coastal, desert, poor and rich Savanah, and forest, its wide open international borders with many neighboring countries, and the high variety and size of domestic and wildlife animal populations in the country make it very prone for the emergence of pandemics of viral hemorrhagic fevers [70]. Additionally, molecular xenosurveillance is a robust tool for the surveillance of emerging infectious diseases and their vectors that leverages the blood-fed vectors as collectors of blood samples from both humans and animals [71,72,73]. Therefore, public health authorities, particularly in poor resource settings, should utilize this approach to reduce the cost of sampling human and animal populations. Particularly, samples of the same species of vectors with similar meta-data (place and date of collection) could be pooled together to further reduce the molecular testing cost. Furthermore, through the implementation of the integrated One Health, molecular xenosurveillance, and response system, there is less delay between identifying diseases circulating in the area and the implementation of control measures to prevent potential epidemics, outbreaks, and pandemics [52,54,72,73]. The added value of this approach, which includes but is not limited to, less morbidity and mortality among humans, less social disturbance, as well as less economic and environmental loss, makes it worth the investment [52,74].

## 4. Materials and Methods

### 4.1. Exploratory Surveys

**Figure 6:**
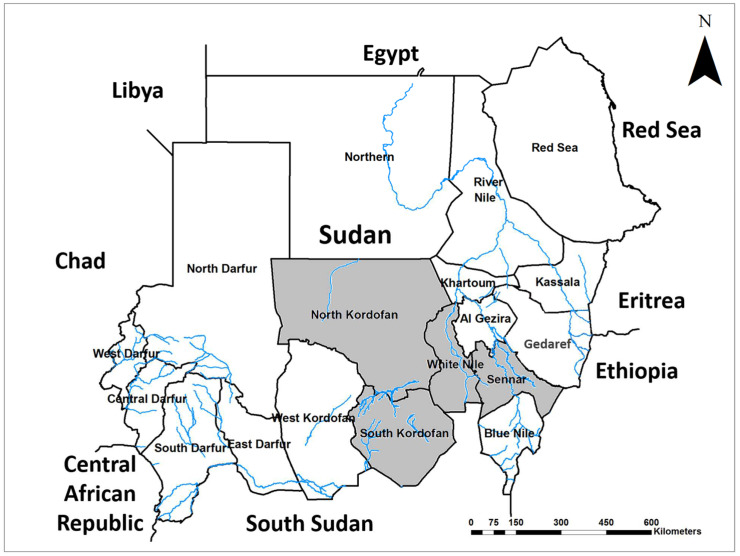
Map of Sudan shows the study area shaded in grey.

In response to the emergence and further spread of the invasive malaria vector, *Anopheles stephensi* in Sudan [20,60], we initiated exploratory surveys targeting to delineate the distribution of *An. stephensi* that co-breed with *Aedes* mosquitoes in human-made water containers [50,75]. Household container surveys confirmed the presence of *An. stephensi* in the eastern, western, and northern international borders; therefore, we focused our second survey on the southern borders of the country, namely North and South Kordofan, Sennar, and White Nile states (Figure 6) [59,75].

### 4.2. Mosquito Samples Collection and Morphological Identification

During the household surveys, we used disposable dippers for the collection of aquatic stages (larvae and pupae) of mosquitoes from the human-made water containers. Then, we transferred the collected larvae to the laboratory at Sirius Training and Research Centre in Khartoum for morphological identification and molecular investigations. We reared the larvae to adults and morphologically identified the adults *Aedes* mosquitoes using standard morphological keys and consulted the online available database of the Walter Reed Biosystematics Unit (WRBU) for further confirmation (https://www.wrbu.si.edu/index.php/) [27,76]. *Anopheles* mosquitoes were identified using the recently published standard key for the morphological identification of Afrotropical *Anopheles* [76].

### 4.3. DNA Extraction from Mosquito and Polymerase Chain Reaction

The genomic DNA was extracted from the mosquito samples following the manufacturer instructions using QiaAmp tissue extraction kits (QIAGEN Diagnostics GmbH, Hilden, Germany). Extracted DNA quality was checked using a nanodrop spectrophotometer (Implen, München, Germany), then preserved at −20 °C until molecular examination.

To amplify the cytochrome oxidase 1 (CO1) region of the mitochondrial DNA of the mosquito genome, the Folmer primers (LCO1490 and HCO2198) were used [77]. PCR reaction mixture containing 2 µL of the extracted DNA was added to a 4 µL PCR master mix (Solis Biodyne, Tartu, Estonia) containing 1 U DNA polymerase, 12.5 mM MgCl2, and 4 mM dNTPs. PCR thermal conditions were as follows: initial denaturation at 95 °C for 5 min, followed by 35 cycles of denaturation at 95 °C for 30 s, annealing at 58 °C for 30 s, and extension at 72 °C for 30 s, and a final extension step at 72 °C for 10 min. Thermal conditions were performed using the 2721 Thermocycler (Applied Biosystems, ThermoFisher Scientific, Budapest, Hungary). Following PCR, amplicons were visualized using 2% gel electrophoresis (Major Science Co., Ltd, Taoyuan City, Taiwan).

### 4.4. PCR Amplicons Sequencing and Sequences’ Identity Confirmation

The amplified PCR amplicons were sequenced in duplicates based on both directions’ primers (LCO1490 and HCO2198) by the Sanger Deoxyribonucleic acid sequencing method using the 3730XL DNA analyzer (Life Technologies Corporation, California, United States) through Macrogen (Macrogen Inc., Amsterdam, The Netherlands). Prior to checking sequences’ identities, sequences of each mosquito sample were aligned on GENtel software to check for any base calling errors during the sequencing process. Following peaks correction, a consensus sequence was constructed from each duplicate. Then, we used the online BLAST nucleotide algorithm to compare our sequences with the previously published *Aedes* species consensus sequences available in the NCBI GenBank database (https://blast.ncbi.nlm.nih.gov/Blast.cgi, accessed on 13 April 2022) [28]. The obtained DNA sequences during this study were deposited into the GenBank database at NCBI.

### 4.5. Bioinformatics and Phylogenetic Analysis

Phylogenetic analysis was conducted using the maximum likelihood method to determine the close relatedness of sequences to the previously published sequences available at the NCBI GenBank database [28]. To construct the phylogenetic tree, all sequences available for the *Ae. albopictus* species were downloaded and each of the data that were related to each sequence were sorted based on country of isolation (Supplementary Materials Table S1). A total of 103 sequences representing African countries were selected for constructing a maximum likelihood phylogenetic tree. The selected sequences and the study sequences were aligned and trimmed to produce similar sequences in length prior to constructing the phylogenetic tree using MEGA7 software. The nucleotide substitution model with the lowest Bayesian Information Criterion (BIC) scores was considered as the best-fit model. The non-uniformity of evolutionary rates among sites was modelled using a discrete Gamma distribution with 1000 bootstraps during the phylogenetic tree construction [78].

Bioinformatics analysis was conducted for all sequences included in the study, including the previously published sequences to obtain sequence diversity parameters, including numbers of haplotypes (H) and haplotypes diversity (Hd) according to each country using the DnaSP v5.10 software [79]. Additionally, a haplotype network was constructed using the median-joining network using popART software (v4.8) (http://popart.otago.ac.nz, accessed on 29 April 2022). Further, to investigate the estimated genetic differentiation between the study sequences and the African countries’ sequences, the pairwise fixation index (Fst) value was calculated using DnaSP [79]. Fst values were interpreted, according to [79], as follows: 0.00–0.05, indicating a small genetic differentiation between the populations; 0.05–0.15, indicating a moderate genetic differentiation among the populations; 0.15–0.25, indicating a large genetic differentiation; and Fst more than 0.25 was indicative of a great genetic differentiation between the populations [79].

### 4.6. Exploring the Potential Health Impacts of the Emergence of This Invasive Vector in the Area

To investigate the potential role of this invasive vector, *Aedes albopictus* and other vectors on the mergence and geographical spread of arboviral disease outbreaks, particularly CHIK in the region, we reviewed the publicly available records about CHIKV outbreaks, particularly rural areas. We compared our findings about outbreaks of relevant *Aedes*-borne arboviruses in the country with the previously mapped distribution of arboviral diseases in Sudan up to 2020 [21].

## 5. Conclusions

Here we provide the first molecular evidence about the emergence of the rural invasive arboviral diseases vector, *Aedes albopictus* in Sudan. This finding was made accidentally; however, this indicates that this vector might be very prevalent in the country. Records about recent outbreaks of chikungunya in the region of East Africa are very alarming due to their massive magnitude and rapid development, and this could be attributed to the contribution of this undetected invasive vector, which is very competent in transmitting several arboviruses, and its adaptive nature to rural areas. An integrated One Health approach is urgently needed in the country to prevent a dire situation in the future, particularly that the country health system is overwhelmingly challenged by the burden of other infectious diseases such as malaria. It would be strategic, less resource-demanding, and less invasive to humans and animals to incorporate molecular xenosurveillance as a proxy for monitoring the dynamics of zoonotic diseases and their vectors in the area.

## Figures and Tables

**Table 1:** Diversity and neutrality indices for *Ae. albopictus* populations in Africa calculated from the nucleotide datasets of the sequences available in the GenBank database at NCBI.

Populations *	N	S	H	Hd ± VarHd	Pi	Tajima’s D	Fu Li’s D	Fu Li’s F
MadagasCAR	12	6	7	0.864 ± 0.00618	0.00321	−1.0217	−1.1084	−1.2312
Algeria	7	1	2	0.286 ± 0.03856	0.00063	−1.0062	−1.0488	−1.1015
CAR	2	2	2	1.0 ± 0.25	0.00442	n.d.	n.d.	n.d.
DRC	45	2	3	0.457 ± 0.00562	0.00129	0.5144	0.7583	0.7967
Mauritius	2	2	2	1.0 ± 0.25	0.00442	n.d.	n.d.	n.d.
Mayotte	3	2	2	0.667 ± 0.09877	0.00294	n.d.	n.d.	n.d.
Cameroon	9	5	6	0.917 ± 0.00526	0.00343	−0.6542	−0.5973	−0.6796
Morocco	2	0	1	0.0 ± 0.00	0.0	n.d.	n.d.	n.d.
R Congo	3	2	3	1.0 ± 0.07407	0.00294	n.d.	n.d.	n.d.
La Réunion	14	3	4	0.648 ± 0.1353	0.0017	−0.5651	0.0168	−0.1524
Sudan	3	0	1	0.0 ± 0.00	0.0	n.d.	n.d.	n.d.
Nigeria	1	0	n.a.	0.0 ± 0.00	0.0	n.d.	n.d.	n.d.
Seychelles	1	0	n.a.	0.0 ± 0.00	0.0	n.d.	n.d.	n.d.
Benin	1	0	n.a.	0.0 ± 0.00	0.0	n.d.	n.d.	n.d.

* CAR: Central African Republic, DRC: Democratic Republic of the Congo, R Congo: Republic of the Congo. N: number of sequences, S: number of segregating sites, H: number of haplotypes, Hd ± VarHd: haplotype diversity ± variance of haplotype diversity, Pi: nucleotide diversity per site. n.a.: not applicable, and n.d.: not determined.

**Table 2:** Pairwise fixation index (Fst test values) between the African populations of *Ae. albopictus* calculated from the nucleotide datasets available in the GenBank.

	DRC	Madagascar	Mauritius	Mayotte	Morocco	R Congo	Sudan	Cameroon	Reunion	CAR
DRC	-	-	-	-	-	-	-	-	-	-
Madagascar	0.613	-	-	-	-	-	-	-	-	-
Mauritius	0.538	0.058	-	-	-	-	-	-	-	-
Mayotte	0.657	**0.004**	0.167	-	-	-	-	-	-	-
Morocco	0.837	0.127	**0.000**	0.333	-	-	-	-	-	-
R Congo	**0.028**	0.442	0.375	0.500	0.600	-	-	-	-	-
Sudan	0.787	0.603	0.500	0.667	1.000	0.667	-	-	-	-
Cameroon	0.051	0.062	0.021	0.178	0.842	0.054	0.662	-	-	-
Reunion	0.648	**0.047**	**0.020**	0.151	0.103	0.462	0.615	0.0721	-	-
CAR	0.456	0.390	0.333	0.444	0.500	0.444	**0.000**	0.493	0.308	-
Algeria	0.776	0.098	**0.000**	0.292	**0.000**	0.553	0.875	0.943	0.077	0.467

CAR: Central African Republic, DRC: Democratic Republic of Congo, and R Congo: Republic of the Congo. Populations where small genetic differentiation is reported were written in bold. Population consisted of only one sequence were excluded: Nigeria, Benin, and Seychelles.

## Data Availability

Sequences of *Ae. albopictus* mosquitoes that were generated during this study were deposited into the GenBank database at NCBI (https://blast.ncbi.nlm.nih.gov/Blast.cgi, accessed 18 April 2022) under the accession numbers ON248551-ON248553. The accession numbers of all *Ae. albopictus* sequences sorted based on country of isolation that were downloaded from the NCBI GenBank database and used in the phylogenetic and haplotype analyses are available in Supplementary Materials Table S1.

## Supplementary Materials

The supporting information can be downloaded at: https://www.mdpi.com/article/10.3390/ijms231911802/s1.
