# Supplementary material for: The First Molecular Detection of Aedes albopictus in Sudan Associates with Increased Outbreaks of Chikungunya and Dengue
Source: Int J Mol Sci. 2022 Oct 5;23(19):11802. doi: 10.3390/ijms231911802 (PMC9570206; doi:10.3390/ijms231911802)
Supplement: Supplementary file 1 [file ijms-23-11802-s001.zip › ijms-1934396-supplementary.pdf]

Supplementary file table S1: The accession numbers of all *Ae. albopictus* sequences that we downloaded from the NCBI GenBank database [28], and sorted based on country of isolation and used in the phylogenetic and haplotype analyses.

**Accession**	**Species**	**Country**
MF148286.1	Ae. albopictus	Malaysia
MF148270.1	Ae. albopictus	Malaysia
MF148288.1	Ae. Albopictus	Malaysia
MF148263.1	Ae. albopictus	Malaysia
MF148261.1	Ae. albopictus	Malaysia
KY982359.1	Ae. albopictus	Malaysia
KY982342.1	Ae. albopictus	Malaysia
KU738424.1	Ae. albopictus	China
KX383926.1	Ae. albopictus	Thailand
MF148287.1	Ae. albopictus	Malaysia
MF148284.1	Ae. albopictus	Malaysia
MF148283.1	Ae. albopictus	Malaysia
MF148265.1	Ae. albopictus	Malaysia
MF148254.1	Ae. albopictus	Malaysia
KY982366.1	Ae. albopictus	Malaysia
KY982364.1	Ae. albopictus	Malaysia
KY982360.1	Ae. albopictus	Malaysia
KY982356.1	Ae. albopictus	Malaysia
KY982355.1	Ae. albopictus	Malaysia
KY982353.1	Ae. albopictus	Malaysia
KY982334.1	Ae. albopictus	Malaysia
KU738429.1	Ae. albopictus	China
KU738425.1	Ae. albopictus	China
KX383934.1	Ae. albopictus	China
KX383928.1	Ae. albopictus	Thailand
KX383927.1	Ae. albopictus	Greece
KX383925.1	Ae. albopictus	Thailand
KX383923.1	Ae. albopictus	Italy
KY982357.1	Ae. albopictus	Malaysia
MN513366.1	Ae. albopictus	Portugal
MN513365.1	Ae. albopictus	Portugal
MN513361.1	Ae. albopictus	Portugal
MN513359.1	Ae. albopictus	Portugal
MN513357.1	Ae. albopictus	Portugal
MN513355.1	Ae. albopictus	Portugal
MN513354.1	Ae. albopictus	Portugal
MN513353.1	Ae. albopictus	Portugal
MN513352.1	Ae. albopictus	Portugal

MF148285.1	Ae. albopictus	Malaysia
MF148282.1	Ae. albopictus	Malaysia
MF148280.1	Ae. albopictus	Malaysia
MF148279.1	Ae. albopictus	Malaysia
MF148268.1	Ae. albopictus	Malaysia
MF148267.1	Ae. albopictus	Malaysia
MF148260.1	Ae. albopictus	Malaysia
MF148258.1	Ae. albopictus	Malaysia
KY982354.1	Ae. albopictus	Malaysia
KY982341.1	Ae. albopictus	Malaysia
KU738427.1	Ae. albopictus	China
KU738407.1	Ae. albopictus	China
KU738386.1	Ae. albopictus	China
KX383933.1	Ae. albopictus	Italy
KX383932.1	Ae. albopictus	Greece
KX383931.1	Ae. albopictus	Albania
KX383930.1	Ae. albopictus	Albania
KX383929.1	Ae. albopictus	Italy
KX383921.1	Ae. albopictus	Italy
KX383916.1	Ae. albopictus	Italy
KR068634.1	Ae. albopictus	China
MN513364.1	Ae. albopictus	Portugal
MF148259.1	Ae. albopictus	Malaysia
MF148252.1	Ae. albopictus	Malaysia
KY982340.1	Ae. albopictus	Malaysia
KY982335.1	Ae. albopictus	Malaysia
KU738430.1	Ae. albopictus	China
KU738408.1	Ae. albopictus	China
KU738406.1	Ae. albopictus	China
KU738401.1	Ae. albopictus	China
KX383920.1	Ae. albopictus	Italy
KX383919.1	Ae. albopictus	USA
KX383918.1	Ae. albopictus	Italy
KX383917.1	Ae. albopictus	USA
KY982336.1	Ae. albopictus	Malaysia
JQ004525.1	Ae. albopictus	Japan
MT993476.1	Ae. albopictus	Greece
KY982362.1	Ae. albopictus	Malaysia
KX809765.1	Ae. albopictus	Japan
MF148255.1	Ae. albopictus	Malaysia
MN513368.1	Ae. albopictus	Portugal
MN513367.1	Ae. albopictus	Portugal
MN513362.1	Ae. albopictus	Portugal

MF148256.1	Ae. albopictus	Malaysia
JQ004524.1	Ae. albopictus	Japan
KX809764.1	Ae. albopictus	Philippines
KX809761.1	Ae. albopictus	Philippines
MK575475.1	Ae. albopictus	Brazil
KY982339.1	Ae. albopictus	Malaysia
KX809763.1	Ae. albopictus	Philippines
KX383935.1	Ae. albopictus	Philippines
NC 006817.1	Ae. albopictus	Taiwan
XR 003893871.1	Ae. albopictus	Italy
KY982337.1	Ae. albopictus	Malaysia
KY971594.1	Ae. albopictus	China
XR 003893569.1	Ae. albopictus	Italy
KY971591.1	Ae. albopictus	China
KY982338.1	Ae. albopictus	Malaysia
MG871396.1	Ae. albopictus	South Korea
MG871393.1	Ae. albopictus	South Korea
MG871378.1	Ae. albopictus	South Korea
KY971599.1	Ae. albopictus	China
KY971596.1	Ae. albopictus	China
KY971592.1	Ae. albopictus	China
KY971602.1	Ae. albopictus	China
MG871401.1	Ae. albopictus	South Korea
MG871400.1	Ae. albopictus	South Korea
MG871399.1	Ae. albopictus	South Korea
MG871398.1	Ae. albopictus	South Korea
MG871397.1	Ae. albopictus	South Korea
MG871395.1	Ae. albopictus	South Korea
MG871394.1	Ae. albopictus	South Korea
MG871392.1	Ae. albopictus	South Korea
MG871365.1	Ae. albopictus	South Korea
KY971598.1	Ae. albopictus	China
MG871391.1	Ae. albopictus	South Korea
MG871390.1	Ae. albopictus	South Korea
MG871388.1	Ae. albopictus	South Korea
MG871386.1	Ae. albopictus	South Korea
MG871384.1	Ae. albopictus	South Korea
MG871383.1	Ae. albopictus	South Korea
MG871382.1	Ae. albopictus	South Korea
MG871381.1	Ae. albopictus	South Korea
MG871380.1	Ae. albopictus	South Korea
MG871379.1	Ae. albopictus	South Korea
MG871377.1	Ae. albopictus	South Korea

MG871376.1	Ae. albopictus	South Korea
MG871375.1	Ae. albopictus	South Korea
MG871374.1	Ae. albopictus	South Korea
MG871373.1	Ae. albopictus	South Korea
MG871372.1	Ae. albopictus	South Korea
MG871371.1	Ae. albopictus	South Korea
MG871370.1	Ae. albopictus	South Korea
MG871369.1	Ae. albopictus	South Korea
MG871368.1	Ae. albopictus	South Korea
MG871367.1	Ae. albopictus	South Korea
MG871366.1	Ae. albopictus	South Korea
KY971593.1	Ae. albopictus	China
MG871402.1	Ae. albopictus	South Korea
MG871389.1	Ae. albopictus	South Korea
MG871387.1	Ae. albopictus	South Korea
MG871385.1	Ae. albopictus	South Korea
KY971601.1	Ae. albopictus	China
KY971597.1	Ae. albopictus	China
KC690922.1	Ae. albopictus	USA
KC690929.1	Ae. albopictus	USA
MW279079.1	Ae. albopictus	Brazil
MW279069.1	Ae. albopictus	Spain
KC690940.1	Ae. albopictus	USA
MW279070.1	Ae. albopictus	Spain
KC690951.1	Ae. albopictus	USA
KC690941.1	Ae. albopictus	USA
KC690935.1	Ae. albopictus	USA
KC690930.1	Ae. albopictus	USA
KC690928.1	Ae. albopictus	USA
KC690924.1	Ae. albopictus	USA
KC690912.1	Ae. albopictus	USA
KC690906.1	Ae. albopictus	USA
MH817501.1	Ae. albopictus	Russia
MF185673.1	Ae. albopictus	Canada
MF185672.1	Ae. albopictus	Canada
MW279074.1	Ae. albopictus	Spain
MW279073.1	Ae. albopictus	Spain
MW279068.1	Ae. albopictus	Spain
KC690961.1	Ae. albopictus	USA
KC690955.1	Ae. albopictus	USA
KC690944.1	Ae. albopictus	USA
KC690933.1	Ae. albopictus	USA
KC690932.1	Ae. albopictus	USA

KC690927.1	Ae. albopictus	USA
KC690925.1	Ae. albopictus	USA
KC690921.1	Ae. albopictus	USA
KC690918.1	Ae. albopictus	USA
KC690917.1	Ae. albopictus	USA
KC690916.1	Ae. albopictus	USA
KC690914.1	Ae. albopictus	USA
KC690910.1	Ae. albopictus	USA
KC690904.1	Ae. albopictus	USA
KC690901.1	Ae. albopictus	USA
KC690898.1	Ae. albopictus	USA
KC690896.1	Ae. albopictus	USA
MF185679.1	Ae. albopictus	USA
MF185670.1	Ae. albopictus	Canada
MF185669.1	Ae. albopictus	Canada
MW279077.1	Ae. albopictus	Spain
MW279076.1	Ae. albopictus	Spain
MW279072.1	Ae. albopictus	Spain
MW279071.1	Ae. albopictus	Spain
KC690959.1	Ae. albopictus	USA
KC690958.1	Ae. albopictus	USA
KC690956.1	Ae. albopictus	USA
KC690953.1	Ae. albopictus	USA
KC690952.1	Ae. albopictus	USA
KC690950.1	Ae. albopictus	USA
KC690949.1	Ae. albopictus	USA
KC690947.1	Ae. albopictus	USA
KC690945.1	Ae. albopictus	USA
KC690943.1	Ae. albopictus	USA
KC690936.1	Ae. albopictus	USA
KC690934.1	Ae. albopictus	USA
KC690923.1	Ae. albopictus	USA
KC690920.1	Ae. albopictus	USA
KC690915.1	Ae. albopictus	USA
KC690913.1	Ae. albopictus	USA
KC690911.1	Ae. albopictus	USA
KC690905.1	Ae. albopictus	USA
KC690903.1	Ae. albopictus	USA
KC690900.1	Ae. albopictus	USA
KC690899.1	Ae. albopictus	USA
KC690897.1	Ae. albopictus	USA
MW776430.1	Ae. albopictus	Russia
MH817538.1	Ae. albopictus	USA

MF185678.1	Ae. albopictus	USA
MW279075.1	Ae. albopictus	Spain
KC690960.1	Ae. albopictus	USA
KC690957.1	Ae. albopictus	USA
KC690954.1	Ae. albopictus	USA
KC690948.1	Ae. albopictus	USA
KC690946.1	Ae. albopictus	USA
KC690942.1	Ae. albopictus	USA
KC690939.1	Ae. albopictus	USA
KC690937.1	Ae. albopictus	USA
KC690931.1	Ae. albopictus	USA
KC690919.1	Ae. albopictus	USA
KC690908.1	Ae. albopictus	USA
KC690907.1	Ae. albopictus	USA
MH817514.1	Ae. albopictus	Russia
MH817523.1	Ae. albopictus	Russia
MF185674.1	Ae. albopictus	Canada
MW279078.1	Ae. albopictus	France
KC690938.1	Ae. albopictus	USA
KC690926.1	Ae. albopictus	USA
KC690909.1	Ae. albopictus	USA
KC690902.1	Ae. albopictus	USA
MW776429.1	Ae. albopictus	Russia
KX383924.1	Ae. albopictus	Brazil
KX383924.1	Ae. albopictus	Brazil
AB907801.1	Ae. albopictus	Panama
AB907796.1	Ae. albopictus	Costa Rica
AB907800.1	Ae. albopictus	Costa Rica
AB907798.1	Ae. albopictus	Costa Rica
AB907797.1	Ae. albopictus	Costa Rica
AB907799.1	Ae. albopictus	Costa Rica
MH817495.1	Ae. albopictus	Russia
MN080743.1	Ae. albopictus	Lao People's Democratic Republic
MN080765.1	Ae. albopictus	Lao People's Democratic Republic
MN080730.1	Ae. albopictus	Lao People's Democratic Republic
MN080729.1	Ae. albopictus	Lao People's Democratic Republic
MH817547.1	Ae. albopictus	Russia
MN080763.1	Ae. albopictus	Lao People's Democratic Republic
MN080761.1	Ae. albopictus	Lao People's Democratic Republic
MN080758.1	Ae. albopictus	Lao People's Democratic Republic
MN080745.1	Ae. albopictus	Lao People's Democratic Republic
MN080740.1	Ae. albopictus	Lao People's Democratic Republic
MN080739.1	Ae. albopictus	Lao People's Democratic Republic

MN080737.1	Ae. albopictus	Lao People's Democratic Republic
MN080736.1	Ae. albopictus	Lao People's Democratic Republic
MN080735.1	Ae. albopictus	Lao People's Democratic Republic
MN080732.1	Ae. albopictus	Lao People's Democratic Republic
MN080731.1	Ae. albopictus	Lao People's Democratic Republic
MN080728.1	Ae. albopictus	Lao People's Democratic Republic
MN080726.1	Ae. albopictus	Lao People's Democratic Republic
MN080724.1	Ae. albopictus	Lao People's Democratic Republic
MN080723.1	Ae. albopictus	Lao People's Democratic Republic
MN080722.1	Ae. albopictus	Lao People's Democratic Republic
MN080720.1	Ae. albopictus	Lao People's Democratic Republic
MH817548.1	Ae. albopictus	Russia
MH817555.1	Ae. albopictus	Russia
MN080764.1	Ae. albopictus	Lao People's Democratic Republic
MN080762.1	Ae. albopictus	Lao People's Democratic Republic
MN080760.1	Ae. albopictus	Lao People's Democratic Republic
MN080759.1	Ae. albopictus	Lao People's Democratic Republic
MN080757.1	Ae. albopictus	Lao People's Democratic Republic
MN080756.1	Ae. albopictus	Lao People's Democratic Republic
MN080755.1	Ae. albopictus	Lao People's Democratic Republic
MN080752.1	Ae. albopictus	Lao People's Democratic Republic
MN080751.1	Ae. albopictus	Lao People's Democratic Republic
MN080750.1	Ae. albopictus	Lao People's Democratic Republic
MN080749.1	Ae. albopictus	Lao People's Democratic Republic
MN080748.1	Ae. albopictus	Lao People's Democratic Republic
MN080747.1	Ae. albopictus	Lao People's Democratic Republic
MN080744.1	Ae. albopictus	Lao People's Democratic Republic
MN080742.1	Ae. albopictus	Lao People's Democratic Republic
MN080741.1	Ae. albopictus	Lao People's Democratic Republic
MN080738.1	Ae. albopictus	Lao People's Democratic Republic
MN080734.1	Ae. albopictus	Lao People's Democratic Republic
MN080727.1	Ae. albopictus	Lao People's Democratic Republic
MN080725.1	Ae. albopictus	Lao People's Democratic Republic
MN080721.1	Ae. albopictus	Lao People's Democratic Republic
MH817537.1	Ae. albopictus	Lao People's Democratic Republic
MN080754.1	Ae. albopictus	Russia
MN080753.1	Ae. albopictus	Lao People's Democratic Republic
MN080746.1	Ae. albopictus	Lao People's Democratic Republic
LC591863.1	Ae. albopictus	Thailand
LC591859.1	Ae. albopictus	Thailand
MN080733.1	Ae. albopictus	Lao People's Democratic Republic
LC591942.1	Ae. albopictus	Japan
LC591898.1	Ae. albopictus	Japan

LC591885.1	Ae. albopictus	Japan
LC591876.1	Ae. albopictus	Japan
LC591870.1	Ae. albopictus	Thailand
LC591869.1	Ae. albopictus	Thailand
LC591866.1	Ae. albopictus	Thailand
LC591862.1	Ae. albopictus	Thailand
LC591860.1	Ae. albopictus	Thailand
LC591925.1	Ae. albopictus	Japan
LC591920.1	Ae. albopictus	Japan
LC591895.1	Ae. albopictus	Japan
LC591887.1	Ae. albopictus	Japan
LC591884.1	Ae. albopictus	Japan
LC591882.1	Ae. albopictus	Japan
LC591881.1	Ae. albopictus	Japan
LC591867.1	Ae. albopictus	Japan
LC591861.1	Ae. albopictus	Thailand
LC597556.1	Ae. albopictus	Japan
LC597554.1	Ae. albopictus	Japan
LC597553.1	Ae. albopictus	Japan
LC597552.1	Ae. albopictus	Japan
LC597551.1	Ae. albopictus	Japan
LC597550.1	Ae. albopictus	Japan
LC591941.1	Ae. albopictus	Japan
LC591940.1	Ae. albopictus	Japan
LC591939.1	Ae. albopictus	Japan
LC591937.1	Ae. albopictus	Japan
LC591936.1	Ae. albopictus	Japan
LC591935.1	Ae. albopictus	Japan
LC591934.1	Ae. albopictus	Japan
LC591933.1	Ae. albopictus	Japan
LC591931.1	Ae. albopictus	Japan
LC591928.1	Ae. albopictus	Japan
LC591924.1	Ae. albopictus	Japan
LC591921.1	Ae. albopictus	Japan
LC591915.1	Ae. albopictus	Japan
LC591914.1	Ae. albopictus	Japan
LC591913.1	Ae. albopictus	Japan
LC591910.1	Ae. albopictus	Japan
LC591909.1	Ae. albopictus	Japan
LC591905.1	Ae. albopictus	Japan
LC591904.1	Ae. albopictus	Japan
LC591903.1	Ae. albopictus	Japan
LC591902.1	Ae. albopictus	Japan

LC591899.1	Ae. albopictus	Japan
LC591894.1	Ae. albopictus	Japan
LC591891.1	Ae. albopictus	Japan
LC591890.1	Ae. albopictus	Japan
LC591879.1	Ae. albopictus	Japan
LC591875.1	Ae. albopictus	Japan
LC591868.1	Ae. albopictus	Thailand
LC591865.1	Ae. albopictus	Thailand
LC591864.1	Ae. albopictus	Thailand
LC591932.1	Ae. albopictus	Japan
LC591930.1	Ae. albopictus	Japan
LC591927.1	Ae. albopictus	Japan
LC591926.1	Ae. albopictus	Japan
LC591923.1	Ae. albopictus	Japan
LC591922.1	Ae. albopictus	Japan
LC591919.1	Ae. albopictus	Japan
LC591918.1	Ae. albopictus	Japan
LC591917.1	Ae. albopictus	Japan
LC591912.1	Ae. albopictus	Japan
LC591911.1	Ae. albopictus	Japan
LC591892.1	Ae. albopictus	Japan
LC591886.1	Ae. albopictus	Japan
LC591880.1	Ae. albopictus	Japan
LC591878.1	Ae. albopictus	Japan
LC591877.1	Ae. albopictus	Japan
MF148271.1	Ae. albopictus	Malaysia
LC597555.1	Ae. albopictus	Japan
LC597549.1	Ae. albopictus	Japan
LC591938.1	Ae. albopictus	Japan
LC591929.1	Ae. albopictus	Japan
LC591916.1	Ae. albopictus	Japan
LC591908.1	Ae. albopictus	Japan
LC591907.1	Ae. albopictus	Japan
LC591906.1	Ae. albopictus	Japan
LC591901.1	Ae. albopictus	Japan
LC591900.1	Ae. albopictus	Japan
LC591897.1	Ae. albopictus	Japan
LC591896.1	Ae. albopictus	Japan
LC591893.1	Ae. albopictus	Japan
LC591889.1	Ae. albopictus	Japan
LC591888.1	Ae. albopictus	Japan
LC591883.1	Ae. albopictus	Japan
LC591872.1	Ae. albopictus	Philippines

LC591871.1	Ae. albopictus	Philippines
LC591874.1	Ae. albopictus	Philippines
MH817521.1	Ae. albopictus	Russia
LC591873.1	Ae. albopictus	Philippines
MZ501561.1	Ae. albopictus	Russia
MZ501560.1	Ae. albopictus	Russia
MZ501555.1	Ae. albopictus	Russia
MZ501554.1	Ae. albopictus	Russia
MZ501552.1	Ae. albopictus	Russia
MZ501540.1	Ae. albopictus	Russia
MZ501537.1	Ae. albopictus	Russia
MZ501518.1	Ae. albopictus	Russia
MZ501517.1	Ae. albopictus	Russia
MZ501516.1	Ae. albopictus	Russia
MZ501510.1	Ae. albopictus	Russia
MZ501508.1	Ae. albopictus	Russia
MH817556.1	Ae. albopictus	Russia
MZ501559.1	Ae. albopictus	Russia
MZ501558.1	Ae. albopictus	Russia
MZ501557.1	Ae. albopictus	Russia
MZ501556.1	Ae. albopictus	Russia
MZ501553.1	Ae. albopictus	Russia
MZ501551.1	Ae. albopictus	Russia
MZ501550.1	Ae. albopictus	Russia
MZ501549.1	Ae. albopictus	Russia
MZ501548.1	Ae. albopictus	Russia
MZ501547.1	Ae. albopictus	Russia
MZ501546.1	Ae. albopictus	Russia
MZ501545.1	Ae. albopictus	Russia
MZ501544.1	Ae. albopictus	Russia
MZ501543.1	Ae. albopictus	Russia
MZ501542.1	Ae. albopictus	Russia
MZ501541.1	Ae. albopictus	Russia
MZ501539.1	Ae. albopictus	Russia
MZ501538.1	Ae. albopictus	Russia
MZ501536.1	Ae. albopictus	Russia
MZ501535.1	Ae. albopictus	Russia
MZ501534.1	Ae. albopictus	Russia
MZ501533.1	Ae. albopictus	Russia
MZ501532.1	Ae. albopictus	Russia
MZ501531.1	Ae. albopictus	Russia
MZ501530.1	Ae. albopictus	Russia
MZ501529.1	Ae. albopictus	Russia

MZ501528.1	Ae. albopictus	Russia
MZ501527.1	Ae. albopictus	Russia
MZ501526.1	Ae. albopictus	Russia
MZ501525.1	Ae. albopictus	Russia
MZ501524.1	Ae. albopictus	Russia
MZ501523.1	Ae. albopictus	Russia
MZ501522.1	Ae. albopictus	Russia
MZ501521.1	Ae. albopictus	Russia
MZ501520.1	Ae. albopictus	Russia
MZ501519.1	Ae. albopictus	Russia
MZ501515.1	Ae. albopictus	Russia
MZ501514.1	Ae. albopictus	Russia
MZ501513.1	Ae. albopictus	Russia
MZ501512.1	Ae. albopictus	Russia
MZ501511.1	Ae. albopictus	Russia
MZ501509.1	Ae. albopictus	Russia
MZ501507.1	Ae. albopictus	Russia
MZ501506.1	Ae. albopictus	Russia
MZ501505.1	Ae. albopictus	Russia
MZ501503.1	Ae. albopictus	Russia
MZ501502.1	Ae. albopictus	Russia
MZ501501.1	Ae. albopictus	Russia
MZ501500.1	Ae. albopictus	Russia
MT711529.1	Ae. albopictus	Russia
MZ501504.1	Ae. albopictus	Russia
KY982346.1	Ae. albopictus	Malaysia
MW283013.1	Ae. albopictus	Nigeria
KY982343.1	Ae. albopictus	Malaysia
KY982348.1	Ae. albopictus	Malaysia
MN513360.1	Ae. albopictus	Portugal
KY982361.1	Ae. albopictus	Malaysia
MF148272.1	Ae. albopictus	Malaysia
MF148262.1	Ae. albopictus	Malaysia
MF148269.1	Ae. albopictus	Malaysia
MF148257.1	Ae. albopictus	Malaysia
KY982344.1	Ae. albopictus	Malaysia
KY982351.1	Ae. albopictus	Malaysia
MF148266.1	Ae. albopictus	Malaysia
KY982345.1	Ae. albopictus	Malaysia
KY982349.1	Ae. albopictus	Malaysia
KY982347.1	Ae. albopictus	Malaysia
MT711530.1	Ae. albopictus	Russia
KY982352.1	Ae. albopictus	Malaysia

KY982365.1	Ae. albopictus	Malaysia
KY982363.1	Ae. albopictus	Malaysia
DQ181458.1	Ae. albopictus	Puerto Rico
KY982350.1	Ae. albopictus	Malaysia
DQ181457.1	Ae. albopictus	Puerto Rico
DQ181451.1	Ae. albopictus	Puerto Rico
DQ397912.1	Ae. albopictus	Japan
DQ397911.1	Ae. albopictus	Japan
DQ397910.1	Ae. albopictus	Japan
DQ397909.1	Ae. albopictus	Indonesia
DQ397908.1	Ae. albopictus	Indonesia
MT755928.1	Ae. albopictus	China
MT755927.1	Ae. albopictus	China
MT188124.2	Ae. albopictus	China
MT188118.2	Ae. albopictus	China
MT188115.2	Ae. albopictus	China
AF253022.1	Ae. albopictus	Italy
MT755929.1	Ae. albopictus	China
MT755925.1	Ae. albopictus	China
MT755924.1	Ae. albopictus	China
MT755921.1	Ae. albopictus	China
MT755918.1	Ae. albopictus	China
MT188116.2	Ae. albopictus	China
MT188114.2	Ae. albopictus	China
MT188111.2	Ae. albopictus	China
GQ143719.1	Ae. albopictus	Australia
MT755926.1	Ae. albopictus	China
MT755923.1	Ae. albopictus	China
MT755922.1	Ae. albopictus	China
MT755920.1	Ae. albopictus	China
MT755919.1	Ae. albopictus	China
MT188129.2	Ae. albopictus	China
MT188128.2	Ae. albopictus	China
MT188127.2	Ae. albopictus	China
MT188125.2	Ae. albopictus	China
MT188123.2	Ae. albopictus	China
MT188121.2	Ae. albopictus	China
MT188120.2	Ae. albopictus	China
MT188119.2	Ae. albopictus	China
MT188117.2	Ae. albopictus	China
MT188113.2	Ae. albopictus	China
MT188112.2	Ae. albopictus	China
MT188130.2	Ae. albopictus	China

MT188126.2	Ae. albopictus	China
MT188122.2	Ae. albopictus	China
KU495082.1	Ae. albopictus	Australia
KP896551.1	Ae. albopictus	Malaysia
KP896575.1	Ae. albopictus	Malaysia
KP896569.1	Ae. albopictus	Malaysia
KP896567.1	Ae. albopictus	Malaysia
KP896559.1	Ae. albopictus	Malaysia
KP896553.1	Ae. albopictus	Malaysia
KP896552.1	Ae. albopictus	Malaysia
KP896550.1	Ae. albopictus	Malaysia
KP896574.1	Ae. albopictus	Malaysia
KP896562.1	Ae. albopictus	Malaysia
KP896558.1	Ae. albopictus	Malaysia
KP896557.1	Ae. albopictus	Malaysia
KP896556.1	Ae. albopictus	Malaysia
KP896555.1	Ae. albopictus	Malaysia
KP896563.1	Ae. albopictus	Malaysia
KP896561.1	Ae. albopictus	Malaysia
MN175498.1	Ae. albopictus	Argentina
KP896565.1	Ae. albopictus	Malaysia
KP877568.1	Ae. albopictus	Colombia
KP877564.1	Ae. albopictus	Colombia
KP896570.1	Ae. albopictus	Malaysia
KP877563.1	Ae. albopictus	Colombia
KP877571.1	Ae. albopictus	Colombia
KP877566.1	Ae. albopictus	Colombia
JQ436996.1	Ae. albopictus	Viet Nam
JQ437007.1	Ae. albopictus	Thailand
JQ437001.1	Ae. albopictus	Bhutan
JQ436998.1	Ae. albopictus	Bhutan
JQ436997.1	Ae. albopictus	Viet Nam
JQ436988.1	Ae. albopictus	China
JQ436984.1	Ae. albopictus	Japan
JQ437006.1	Ae. albopictus	Thailand
JQ437005.1	Ae. albopictus	Thailand
JQ437004.1	Ae. albopictus	Thailand
JQ437003.1	Ae. albopictus	Thailand
JQ437002.1	Ae. albopictus	Bhutan
JQ437000.1	Ae. albopictus	Bhutan
JQ436999.1	Ae. albopictus	Bhutan
JQ436995.1	Ae. albopictus	Viet Nam
JQ436991.1	Ae. albopictus	China

JQ436990.1	Ae. albopictus	China
JQ436989.1	Ae. albopictus	China
JQ436987.1	Ae. albopictus	Japan
JQ436985.1	Ae. albopictus	Japan
JQ436982.1	Ae. albopictus	Japan
JQ436981.1	Ae. albopictus	Japan
MK372914.1	Ae. albopictus	USA
JQ437008.1	Ae. albopictus	Thailand
JQ436994.1	Ae. albopictus	Viet Nam
JQ436993.1	Ae. albopictus	Viet Nam
JQ436992.1	Ae. albopictus	Viet Nam
JQ436986.1	Ae. albopictus	Japan
JQ436983.1	Ae. albopictus	Japan
KX266669.1	Ae. albopictus	China
KJ410335.1	Ae. albopictus	India
JQ388786.1	Ae. albopictus	Germany
MW829500.1	Ae. albopictus	South Korea
KX266726.1	Ae. albopictus	China
KX266694.1	Ae. albopictus	China
KX266693.1	Ae. albopictus	China
MK714009.1	Ae. albopictus	Turkey
MK714008.1	Ae. albopictus	Turkey
MK714006.1	Ae. albopictus	Turkey
MK713991.1	Ae. albopictus	Turkey
KX266725.1	Ae. albopictus	China
KX266723.1	Ae. albopictus	China
KX266720.1	Ae. albopictus	China
KX266714.1	Ae. albopictus	China
KX266709.1	Ae. albopictus	China
KX266677.1	Ae. albopictus	China
KX266665.1	Ae. albopictus	China
KX266653.1	Ae. albopictus	China
KX266649.1	Ae. albopictus	China
KX266647.1	Ae. albopictus	China
KX266643.1	Ae. albopictus	China
KX266642.1	Ae. albopictus	China
KX266639.1	Ae. albopictus	China
KX266638.1	Ae. albopictus	China
KF564663.1	Ae. albopictus	Singapore
KF564662.1	Ae. albopictus	Singapore
MW542315.1	Ae. albopictus	India
KX266719.1	Ae. albopictus	China
MW940859.1	Ae. albopictus	China

MT890465.1	Ae. albopictus	China
KX266660.1	Ae. albopictus	China
KX266658.1	Ae. albopictus	China
MZ007511.1	Ae. albopictus	China
MT906350.1	Ae. albopictus	China
MK714010.1	Ae. albopictus	Turkey
MK372913.1	Ae. albopictus	USA
KX266721.1	Ae. albopictus	China
KX266718.1	Ae. albopictus	China
KX266717.1	Ae. albopictus	China
KX266690.1	Ae. albopictus	China
KX266679.1	Ae. albopictus	China
KX266675.1	Ae. albopictus	China
KX266656.1	Ae. albopictus	China
KX266646.1	Ae. albopictus	China
KX266641.1	Ae. albopictus	China
KF564664.1	Ae. albopictus	Singapore
KJ410333.1	Ae. albopictus	India
MW940866.1	Ae. albopictus	China
JX679374.1	Ae. albopictus	Italy
MT890489.1	Ae. albopictus	China
MK542378.1	Ae. albopictus	India
KX266710.1	Ae. albopictus	China
KX266645.1	Ae. albopictus	China
OK266909.1	Ae. albopictus	Ecuador
MZ007515.1	Ae. albopictus	China
MZ007513.1	Ae. albopictus	China
MZ007512.1	Ae. albopictus	China
MW940871.1	Ae. albopictus	China
MT906351.1	Ae. albopictus	China
MT906352.1	Ae. albopictus	China
KY817524.1	Ae. albopictus	Malaysia
KX266670.1	Ae. albopictus	China
KX266666.1	Ae. albopictus	China
KX266664.1	Ae. albopictus	China
KX266640.1	Ae. albopictus	China
OK266901.1	Ae. albopictus	Ecuador
MT906353.1	Ae. albopictus	China
MT906156.1	Ae. albopictus	China
MT890464.1	Ae. albopictus	China
MG242466.1	Ae. albopictus	USA
KX266697.1	Ae. albopictus	China
KX266672.1	Ae. albopictus	China

OK266908.1	Ae. albopictus	Ecuador
MZ007514.1	Ae. albopictus	China
JX679377.1	Ae. albopictus	Italy
JX679373.1	Ae. albopictus	Italy
KX266667.1	Ae. albopictus	China
MW321941.1	Ae. albopictus	Singapore
KX266712.1	Ae. albopictus	China
OK266912.1	Ae. albopictus	Ecuador
OK266899.1	Ae. albopictus	Ecuador
MW321942.1	Ae. albopictus	Singapore
MK297326.1	Ae. albopictus	India
KX266685.1	Ae. albopictus	China
KX266680.1	Ae. albopictus	China
KX266671.1	Ae. albopictus	China
KX266668.1	Ae. albopictus	China
MW457633.1	Ae. albopictus	Austria
KX266700.1	Ae. albopictus	China
OK266904.1	Ae. albopictus	Ecuador
JQ235749.1	Ae. albopictus	China
KX266682.1	Ae. albopictus	China
OK266944.1	Ae. albopictus	Ecuador
OK266925.1	Ae. albopictus	Ecuador
OK266914.1	Ae. albopictus	Ecuador
OK266910.1	Ae. albopictus	Ecuador
KX266678.1	Ae. albopictus	China
OK266947.1	Ae. albopictus	Ecuador
OK266945.1	Ae. albopictus	Ecuador
OK266931.1	Ae. albopictus	Ecuador
OK266930.1	Ae. albopictus	Ecuador
OK266927.1	Ae. albopictus	Ecuador
OK266926.1	Ae. albopictus	Ecuador
OK266921.1	Ae. albopictus	Ecuador
OK266917.1	Ae. albopictus	Ecuador
OK266915.1	Ae. albopictus	Ecuador
OK266913.1	Ae. albopictus	Ecuador
OK266907.1	Ae. albopictus	Ecuador
OK266905.1	Ae. albopictus	Ecuador
OK266900.1	Ae. albopictus	Ecuador
MW321940.1	Ae. albopictus	Singapore
MK182262.1	Ae. albopictus	Pakistan
MN103394.1	Ae. albopictus	Austria
KX266708.1	Ae. albopictus	China
OK266948.1	Ae. albopictus	Ecuador

OK266943.1	Ae. albopictus	Ecuador
OK266939.1	Ae. albopictus	Ecuador
OK266938.1	Ae. albopictus	Ecuador
OK266923.1	Ae. albopictus	Ecuador
OK266916.1	Ae. albopictus	Ecuador
OK266911.1	Ae. albopictus	Ecuador
OK266903.1	Ae. albopictus	Ecuador
MN299017.1	Ae. albopictus	Democratic Republic of the Congo
OK266932.1	Ae. albopictus	Ecuador
OK266929.1	Ae. albopictus	Ecuador
OK266928.1	Ae. albopictus	Ecuador
OK266920.1	Ae. albopictus	Ecuador
OK266919.1	Ae. albopictus	Ecuador
OK266918.1	Ae. albopictus	Ecuador
OK266906.1	Ae. albopictus	Ecuador
OK266902.1	Ae. albopictus	Ecuador
MN103393.1	Ae. albopictus	Austria
KX266722.1	Ae. albopictus	China
KX266706.1	Ae. albopictus	China
OK266935.1	Ae. albopictus	Ecuador
OK266933.1	Ae. albopictus	Ecuador
MN997608.1	Ae. albopictus	Colombia
OK266946.1	Ae. albopictus	Ecuador
OK266941.1	Ae. albopictus	Ecuador
OK266936.1	Ae. albopictus	Ecuador
MT345358.1	Ae. albopictus	Democratic Republic of the Congo
MN299019.1	Ae. albopictus	USA
MK995332.1	Ae. albopictus	Portugal
OK266940.1	Ae. albopictus	Ecuador
OK266937.1	Ae. albopictus	Ecuador
OK266934.1	Ae. albopictus	Ecuador
OK266922.1	Ae. albopictus	Ecuador
KX266657.1	Ae. albopictus	China
OK266949.1	Ae. albopictus	Ecuador
MT345357.1	Ae. albopictus	Democratic Republic of the Congo
MZ573312.1	Ae. albopictus	Viet Nam
MT345356.1	Ae. albopictus	Democratic Republic of the Congo
MT345355.1	Ae. albopictus	Democratic Republic of the Congo
MT345354.1	Ae. albopictus	Democratic Republic of the Congo
MT345353.1	Ae. albopictus	Democratic Republic of the Congo
MT345352.1	Ae. albopictus	Democratic Republic of the Congo
MT345351.1	Ae. albopictus	Democratic Republic of the Congo
HQ398900.1	Ae. albopictus	Viet Nam

MF990905.1	Ae. albopictus	Portugal
MZ573373.1	Ae. albopictus	Viet Nam
MH330191.1	Ae. albopictus	Sri Lanka
DQ424959.1	Ae. albopictus	India
KX266724.1	Ae. albopictus	China
KX266699.1	Ae. albopictus	China
KX266662.1	Ae. albopictus	China
KF406415.1	Ae. albopictus	Pakistan
MN997607.1	Ae. albopictus	Colombia
MN997606.1	Ae. albopictus	Colombia
MN005056.1	Ae. albopictus	Greece
MN005055.1	Ae. albopictus	Greece
MN005054.1	Ae. albopictus	Greece
MG921178.1	Ae. albopictus	Laos
KP843396.1	Ae. albopictus	Thailand
MT999327.1	Ae. albopictus	Mexico
MT999274.1	Ae. albopictus	Mexico
MT999263.1	Ae. albopictus	Mexico
MT999219.1	Ae. albopictus	Mexico
OK465207.1	Ae. albopictus	China
OK465206.1	Ae. albopictus	China
OK465205.1	Ae. albopictus	China
MZ573375.1	Ae. albopictus	Viet Nam
MZ573374.1	Ae. albopictus	Viet Nam
MZ573370.1	Ae. albopictus	Viet Nam
MZ573368.1	Ae. albopictus	Viet Nam
MZ573367.1	Ae. albopictus	Viet Nam
MZ573363.1	Ae. albopictus	Viet Nam
MZ573361.1	Ae. albopictus	Viet Nam
MZ573357.1	Ae. albopictus	Viet Nam
MZ573356.1	Ae. albopictus	Viet Nam
MZ573354.1	Ae. albopictus	Viet Nam
MZ573353.1	Ae. albopictus	Viet Nam
MZ573351.1	Ae. albopictus	Viet Nam
MZ573343.1	Ae. albopictus	Viet Nam
MZ573324.1	Ae. albopictus	Viet Nam
MZ573323.1	Ae. albopictus	Viet Nam
MZ573319.1	Ae. albopictus	Viet Nam
MZ573318.1	Ae. albopictus	Viet Nam
MZ573317.1	Ae. albopictus	Viet Nam
MZ573316.1	Ae. albopictus	Viet Nam
MZ573315.1	Ae. albopictus	Viet Nam
MZ573314.1	Ae. albopictus	Viet Nam

MT552542.1	Ae. albopictus	Mexico
MT552470.1	Ae. albopictus	Mexico
MW961317.1	Ae. albopictus	Portugal
MW961316.1	Ae. albopictus	Portugal
MW961315.1	Ae. albopictus	Portugal
MZ230340.1	Ae. albopictus	Viet Nam
MZ230339.1	Ae. albopictus	Viet Nam
MZ230338.1	Ae. albopictus	Viet Nam
MZ230337.1	Ae. albopictus	Russia
MK505606.1	Ae. albopictus	Montenegro
MK505602.1	Ae. albopictus	Malaysia
MK505594.1	Ae. albopictus	Montenegro
MK505592.1	Ae. albopictus	Montenegro
MK505589.1	Ae. albopictus	Montenegro
MK505583.1	Ae. albopictus	Montenegro
MK505580.1	Ae. albopictus	Montenegro
MK505573.1	Ae. albopictus	Malaysia
MK505570.1	Ae. albopictus	Montenegro
MT345384.1	Ae. albopictus	Democratic Republic of the Congo
MT345383.1	Ae. albopictus	Democratic Republic of the Congo
MT345382.1	Ae. albopictus	Democratic Republic of the Congo
MT345381.1	Ae. albopictus	Democratic Republic of the Congo
MT345380.1	Ae. albopictus	Democratic Republic of the Congo
MT345379.1	Ae. albopictus	Democratic Republic of the Congo
MT345378.1	Ae. albopictus	Democratic Republic of the Congo
MT345377.1	Ae. albopictus	Democratic Republic of the Congo
MT345376.1	Ae. albopictus	Democratic Republic of the Congo
MT345375.1	Ae. albopictus	Democratic Republic of the Congo
MT345374.1	Ae. albopictus	Democratic Republic of the Congo
MT345373.1	Ae. albopictus	Democratic Republic of the Congo
MT345372.1	Ae. albopictus	Democratic Republic of the Congo
MT345371.1	Ae. albopictus	Democratic Republic of the Congo
MT345370.1	Ae. albopictus	Democratic Republic of the Congo
MT345369.1	Ae. albopictus	Democratic Republic of the Congo
MT345368.1	Ae. albopictus	Democratic Republic of the Congo
MT345367.1	Ae. albopictus	Democratic Republic of the Congo
AB690835.1	Ae. albopictus	Japan
MK995331.1	Ae. albopictus	Portugal
MK995326.1	Ae. albopictus	Portugal
MK995319.1	Ae. albopictus	Portugal
MG572237.1	Ae. albopictus	Bangladesh
KX266663.1	Ae. albopictus	China
MZ573322.1	Ae. albopictus	Viet Nam

MH330190.1	Ae. albopictus	Sri Lanka
MK736660.1	Ae. albopictus	India
KX266689.1	Ae. albopictus	China
KX266688.1	Ae. albopictus	China
KM457533.1	Ae. albopictus	China
MT345363.1	Ae. albopictus	Democratic Republic of the Congo
MT345362.1	Ae. albopictus	Democratic Republic of the Congo
MT345361.1	Ae. albopictus	Democratic Republic of the Congo
MT345350.1	Ae. albopictus	Democratic Republic of the Congo
KU319450.1	Ae. albopictus	Spain
KU319448.1	Ae. albopictus	Spain
KU319447.1	Ae. albopictus	Spain
KU319444.1	Ae. albopictus	Spain
KY825745.1	Ae. albopictus	China
KR349286.1	Ae. albopictus	China
KR349284.1	Ae. albopictus	China
KT358457.1	Ae. albopictus	South Korea
KM457544.1	Ae. albopictus	The Netherlands
KM457542.1	Ae. albopictus	The Netherlands
KM457529.1	Ae. albopictus	The Netherlands
MT999330.1	Ae. albopictus	Mexico
MT999164.1	Ae. albopictus	Mexico
MZ573376.1	Ae. albopictus	Viet Nam
MZ573372.1	Ae. albopictus	Viet Nam
MZ573371.1	Ae. albopictus	Viet Nam
MZ573366.1	Ae. albopictus	Viet Nam
MZ573365.1	Ae. albopictus	Viet Nam
MZ573364.1	Ae. albopictus	Viet Nam
MZ573362.1	Ae. albopictus	Viet Nam
MZ573359.1	Ae. albopictus	Viet Nam
MZ573352.1	Ae. albopictus	Viet Nam
MZ573350.1	Ae. albopictus	Viet Nam
MZ573349.1	Ae. albopictus	Viet Nam
MZ573345.1	Ae. albopictus	Viet Nam
MZ573344.1	Ae. albopictus	Viet Nam
MZ573341.1	Ae. albopictus	Viet Nam
MZ573340.1	Ae. albopictus	Viet Nam
MZ573339.1	Ae. albopictus	Viet Nam
MZ573338.1	Ae. albopictus	Viet Nam
MZ573336.1	Ae. albopictus	Viet Nam
MZ573335.1	Ae. albopictus	Viet Nam
MZ573334.1	Ae. albopictus	Viet Nam
MZ573327.1	Ae. albopictus	Viet Nam

MZ573326.1	Ae. albopictus	Viet Nam
MZ573325.1	Ae. albopictus	Viet Nam
MZ573320.1	Ae. albopictus	Viet Nam
MT552522.1	Ae. albopictus	Mexico
MT552484.1	Ae. albopictus	Viet Nam
MT552393.1	Ae. albopictus	Viet Nam
MZ230336.1	Ae. albopictus	Ukraine
MK505609.1	Ae. albopictus	Mexico
MK505575.1	Ae. albopictus	Montenegro
AB738197.1	Ae. albopictus	Japan
AB738161.1	Ae. albopictus	Japan
AB738090.1	Ae. albopictus	Japan
HQ398901.1	Ae. albopictus	Viet Nam
MK284525.1	Ae. albopictus	India
KX886304.1	Ae. albopictus	China
KM457549.1	Ae. albopictus	The Netherlands
MH330193.1	Ae. albopictus	Sri Lanka
MH330192.1	Ae. albopictus	Sri Lanka
MH330189.1	Ae. albopictus	Sri Lanka
MT345386.1	Ae. albopictus	Democratic Republic of the Congo
MT345366.1	Ae. albopictus	Democratic Republic of the Congo
MT345365.1	Ae. albopictus	Democratic Republic of the Congo
MT345364.1	Ae. albopictus	Democratic Republic of the Congo
MT345359.1	Ae. albopictus	Democratic Republic of the Congo
KX886338.1	Ae. albopictus	Democratic Republic of the Congo
KU319449.1	Ae. albopictus	Spain
KM497420.1	Ae. albopictus	China
KM457548.1	Ae. albopictus	The Netherlands
KF406512.1	Ae. albopictus	Pakistan
KF406478.1	Ae. albopictus	Pakistan
KF406398.1	Ae. albopictus	Pakistan
MZ573369.1	Ae. albopictus	Viet Nam
MZ573360.1	Ae. albopictus	Viet Nam
MZ573358.1	Ae. albopictus	Viet Nam
MZ573342.1	Ae. albopictus	Viet Nam
MZ573337.1	Ae. albopictus	Viet Nam
MZ573333.1	Ae. albopictus	Viet Nam
MZ573332.1	Ae. albopictus	Viet Nam
MZ573331.1	Ae. albopictus	Viet Nam
MZ573330.1	Ae. albopictus	Viet Nam
MZ573329.1	Ae. albopictus	Viet Nam
MZ573328.1	Ae. albopictus	Viet Nam
MZ573321.1	Ae. albopictus	Viet Nam

MZ573313.1	Ae. albopictus	Viet Nam
MW228485.1	Ae. albopictus	China
KY765473.1	Ae. albopictus	China
KX886337.1	Ae. albopictus	China
KX886336.1	Ae. albopictus	China
KX886334.1	Ae. albopictus	China
KX886331.1	Ae. albopictus	China
KX886320.1	Ae. albopictus	China
KX886317.1	Ae. albopictus	China
KX886314.1	Ae. albopictus	China
KX886312.1	Ae. albopictus	China
KX886311.1	Ae. albopictus	China
KX886310.1	Ae. albopictus	China
KX886307.1	Ae. albopictus	China
KX886306.1	Ae. albopictus	China
KX886305.1	Ae. albopictus	China
KX886284.1	Ae. albopictus	China
KR817732.1	Ae. albopictus	India
KF406435.1	Ae. albopictus	Pakistan
OK266942.1	Ae. albopictus	Ecuador
OK266924.1	Ae. albopictus	Ecuador
MK505590.1	Ae. albopictus	Malaysia
MN299018.1	Ae. albopictus	Democratic Republic of the Congo
MH578169.1	Ae. albopictus	China
OM131686.1	Ae. albopictus	Japan
OM131682.1	Ae. albopictus	Japan
OM131681.1	Ae. albopictus	Japan
KF406545.1	Ae. albopictus	Pakistan
KF406491.1	Ae. albopictus	Pakistan
OK465352.1	Ae. albopictus	China
OK465337.1	Ae. albopictus	China
OK465334.1	Ae. albopictus	China
OK465331.1	Ae. albopictus	China
OK465330.1	Ae. albopictus	China
OK465323.1	Ae. albopictus	China
OK465322.1	Ae. albopictus	China
OK465321.1	Ae. albopictus	China
OK465312.1	Ae. albopictus	China
OK465310.1	Ae. albopictus	China
OK465309.1	Ae. albopictus	China
OK465308.1	Ae. albopictus	China
OK465297.1	Ae. albopictus	China
OK465296.1	Ae. albopictus	China

OK465289.1	Ae. albopictus	China
OK465288.1	Ae. albopictus	China
OK465281.1	Ae. albopictus	China
OK465280.1	Ae. albopictus	China
OK465279.1	Ae. albopictus	China
OK465268.1	Ae. albopictus	China
OK465267.1	Ae. albopictus	China
OK465262.1	Ae. albopictus	China
OK465254.1	Ae. albopictus	China
MW228484.1	Ae. albopictus	China
KF406540.1	Ae. albopictus	Pakistan
KF406523.1	Ae. albopictus	Pakistan
KF406495.1	Ae. albopictus	Pakistan
KF406472.1	Ae. albopictus	Pakistan
KF406445.1	Ae. albopictus	Pakistan
KF406407.1	Ae. albopictus	Pakistan
KF406404.1	Ae. albopictus	Pakistan
KC970275.1	Ae. albopictus	India
OK465351.1	Ae. albopictus	China
MZ573355.1	Ae. albopictus	Viet Nam
MZ573348.1	Ae. albopictus	Viet Nam
MZ573347.1	Ae. albopictus	Viet Nam
MW228483.1	Ae. albopictus	China
MK439903.1	Ae. albopictus	Czech Republic
MG913592.1	Ae. albopictus	Bangladesh
KY765503.1	Ae. albopictus	China
KY765502.1	Ae. albopictus	China
KY765500.1	Ae. albopictus	China
KY765484.1	Ae. albopictus	China
KY765483.1	Ae. albopictus	China
KY765474.1	Ae. albopictus	China
KX886341.1	Ae. albopictus	China
KX886335.1	Ae. albopictus	China
KX886332.1	Ae. albopictus	China
KX886328.1	Ae. albopictus	China
KX886323.1	Ae. albopictus	China
KX886319.1	Ae. albopictus	China
KX886316.1	Ae. albopictus	China
KX886315.1	Ae. albopictus	China
KX886313.1	Ae. albopictus	China
KX886309.1	Ae. albopictus	China
KX886298.1	Ae. albopictus	China
KX886296.1	Ae. albopictus	China

KX886295.1	Ae. albopictus	China
KX886294.1	Ae. albopictus	China
KX886293.1	Ae. albopictus	China
KX886290.1	Ae. albopictus	China
KX886289.1	Ae. albopictus	China
KX886288.1	Ae. albopictus	China
KX886286.1	Ae. albopictus	China
KX886285.1	Ae. albopictus	China
KX886325.1	Ae. albopictus	China
LC054326.1	Ae. albopictus	Japan
LC054323.1	Ae. albopictus	Japan
OK465340.1	Ae. albopictus	China
MT345387.1	Ae. albopictus	Democratic Republic of the Congo
MZ573346.1	Ae. albopictus	Viet Nam
MW702546.1	Ae. albopictus	spain
MN514831.1	Ae. albopictus	USA
KY765499.1	Ae. albopictus	China
KY765482.1	Ae. albopictus	China
KY765481.1	Ae. albopictus	China
KY765458.1	Ae. albopictus	China
KY765457.1	Ae. albopictus	China
KY765456.1	Ae. albopictus	China
KX886333.1	Ae. albopictus	China
KX886330.1	Ae. albopictus	China
KX886327.1	Ae. albopictus	China
KX886326.1	Ae. albopictus	China
KX886322.1	Ae. albopictus	China
KX886321.1	Ae. albopictus	China
KX886318.1	Ae. albopictus	China
KX886297.1	Ae. albopictus	China
KX886291.1	Ae. albopictus	China
KX886287.1	Ae. albopictus	China
MH025950.1	Ae. albopictus	Republic of the Congo
OM131689.1	Ae. albopictus	Japan
OM131688.1	Ae. albopictus	Japan
OM131685.1	Ae. albopictus	Japan
OM131684.1	Ae. albopictus	Japan
OM131683.1	Ae. albopictus	Japan
OM131680.1	Ae. albopictus	Japan
OM131679.1	Ae. albopictus	Japan
OM131675.1	Ae. albopictus	Japan
OK465313.1	Ae. albopictus	China
OK465311.1	Ae. albopictus	China

MT345385.1	Ae. albopictus	Democratic Republic of the Congo
MG921177.1	Ae. albopictus	Laos
MT434292.1	Ae. albopictus	Viet Nam
MH921569.1	Ae. albopictus	Cameroon
KX981868.1	Ae. albopictus	China
KX886340.1	Ae. albopictus	China
KX886339.1	Ae. albopictus	China
KM613097.1	Ae. albopictus	Thailand
KF406431.1	Ae. albopictus	Pakistan
MN509201.1	Ae. albopictus	USA
LC431230.1	Ae. albopictus	spain
MH025949.1	Ae. albopictus	Republic of the Congo
OM131678.1	Ae. albopictus	Japan
KF406446.1	Ae. albopictus	Pakistan
MT345388.1	Ae. albopictus	Democratic Republic of the Congo
MH921570.1	Ae. albopictus	Cameroon
KU522419.1	Ae. albopictus	Morocco
KM613107.1	Ae. albopictus	Thailand
KM613099.1	Ae. albopictus	Thailand
MW577640.1	Ae. albopictus	Algeria
MH025948.1	Ae. albopictus	Republic of the Congo
KU522420.1	Ae. albopictus	Morocco
KF406563.1	Ae. albopictus	Pakistan
KF406546.1	Ae. albopictus	Pakistan
MT434293.1	Ae. albopictus	Viet Nam
MK714003.1	Ae. albopictus	Turkey
MK598757.1	Ae. albopictus	India
LC431229.1	Ae. albopictus	spain
OK465243.1	Ae. albopictus	China
MH921571.1	Ae. albopictus	Cameroon
MH921568.1	Ae. albopictus	Cameroon
KX886292.1	Ae. albopictus	China
KM613092.1	Ae. albopictus	Thailand
LC428095.1	Ae. albopictus	spain
MH921572.1	Ae. albopictus	Cameroon
KX886329.1	Ae. albopictus	China
MH330188.1	Ae. albopictus	Sri Lanka
LC428094.1	Ae. albopictus	spain
KF406509.1	Ae. albopictus	Pakistan
EU259306.1	Ae. albopictus	India
KY817536.1	Ae. albopictus	Malaysia
KX266695.1	Ae. albopictus	China
KF406567.1	Ae. albopictus	Pakistan

KF406467.1	Ae. albopictus	Pakistan
KY817525.1	Ae. albopictus	Malaysia
KX981867.1	Ae. albopictus	China
KF406453.1	Ae. albopictus	Pakistan
KF406430.1	Ae. albopictus	Pakistan
MN509206.1	Ae. albopictus	USA
OK090993.1	Ae. albopictus	Benin
KT211235.1	Ae. albopictus	Malaysia
KY817565.1	Ae. albopictus	Malaysia
KF406476.1	Ae. albopictus	Pakistan
KF406568.1	Ae. albopictus	Pakistan
KF406483.1	Ae. albopictus	Pakistan
XR 003892638.1	Ae. albopictus	Italy
KT211242.1	Ae. albopictus	Malaysia
KT211240.1	Ae. albopictus	Malaysia
KT211237.1	Ae. albopictus	Malaysia
KT211236.1	Ae. albopictus	Malaysia
KT211233.1	Ae. albopictus	Malaysia
KT211230.1	Ae. albopictus	Malaysia
KT211227.1	Ae. albopictus	Malaysia
KT211221.1	Ae. albopictus	Malaysia
KF406481.1	Ae. albopictus	Pakistan
KY817563.1	Ae. albopictus	Malaysia
KF406473.1	Ae. albopictus	Pakistan
KF406484.1	Ae. albopictus	Pakistan
KT211241.1	Ae. albopictus	Pakistan
KT211239.1	Ae. albopictus	Pakistan
KT211238.1	Ae. albopictus	Pakistan
KT211231.1	Ae. albopictus	Pakistan
KT211229.1	Ae. albopictus	Pakistan
KT211228.1	Ae. albopictus	Pakistan
KT211226.1	Ae. albopictus	Pakistan
KT211225.1	Ae. albopictus	Pakistan
KU926300.1	Ae. albopictus	Mauritius
KF406458.1	Ae. albopictus	Pakistan
KY817535.1	Ae. albopictus	Malaysia
KF406436.1	Ae. albopictus	Pakistan
OL437325.1	Ae. albopictus	USA
MW577638.1	Ae. albopictus	Algeria
KT211234.1	Ae. albopictus	Malaysia
KT211232.1	Ae. albopictus	Malaysia
KT211224.1	Ae. albopictus	Malaysia
KT211223.1	Ae. albopictus	Malaysia

KF406485.1	Ae. albopictus	Pakistan
JF810659.1	Ae. albopictus	Greece
KU926301.1	Ae. albopictus	Italy
KF406561.1	Ae. albopictus	Pakistan
KF406456.1	Ae. albopictus	Pakistan
MK714007.1	Ae. albopictus	Turkey
KF406529.1	Ae. albopictus	Pakistan
KF406482.1	Ae. albopictus	Pakistan
KF406429.1	Ae. albopictus	Pakistan
KT211222.1	Ae. albopictus	Malaysia
MG198595.2	Ae. albopictus	Russia
KF406451.1	Ae. albopictus	Pakistan
KF406433.1	Ae. albopictus	Pakistan
MK429687.1	Ae. albopictus	Italy
MT541179.1	Ae. albopictus	China
KM457524.1	Ae. albopictus	The Netherlands
KM502245.1	Ae. albopictus	China
MG198601.2	Ae. albopictus	Russia
KF406442.1	Ae. albopictus	Pakistan
MK429679.1	Ae. albopictus	Italy
KX886324.1	Ae. albopictus	China
KF406578.1	Ae. albopictus	Pakistan
MT541439.1	Ae. albopictus	China
MT541416.1	Ae. albopictus	China
MT541400.1	Ae. albopictus	China
MT541399.1	Ae. albopictus	China
MT541394.1	Ae. albopictus	China
MT541377.1	Ae. albopictus	China
MT541308.1	Ae. albopictus	China
MT541235.1	Ae. albopictus	China
MT541208.1	Ae. albopictus	China
MT541198.1	Ae. albopictus	China
MT541189.1	Ae. albopictus	China
MT541188.1	Ae. albopictus	China
MT541165.1	Ae. albopictus	China
MT541161.1	Ae. albopictus	China
KM502239.1	Ae. albopictus	China
MK429690.1	Ae. albopictus	Italy
MW577645.1	Ae. albopictus	Algeria
MW577643.1	Ae. albopictus	Algeria
MK429692.1	Ae. albopictus	Italy
MK429680.1	Ae. albopictus	Italy
MT541477.1	Ae. albopictus	China

MT541474.1	Ae. albopictus	China
MT541448.1	Ae. albopictus	China
MT541443.1	Ae. albopictus	China
MT541442.1	Ae. albopictus	China
MT541424.1	Ae. albopictus	China
MT541407.1	Ae. albopictus	China
MT541404.1	Ae. albopictus	China
MT541398.1	Ae. albopictus	China
MT541384.1	Ae. albopictus	China
MT541380.1	Ae. albopictus	China
MT541370.1	Ae. albopictus	China
MT541365.1	Ae. albopictus	China
MT541310.1	Ae. albopictus	China
MT541286.1	Ae. albopictus	China
MT541275.1	Ae. albopictus	China
MT541267.1	Ae. albopictus	China
MT541261.1	Ae. albopictus	China
MT541246.1	Ae. albopictus	China
MT541236.1	Ae. albopictus	China
MT541233.1	Ae. albopictus	China
MT541230.1	Ae. albopictus	China
MT541224.1	Ae. albopictus	China
MT541181.1	Ae. albopictus	China
MH885495.1	Ae. albopictus	Bangladesh
KM502241.1	Ae. albopictus	China
KF406527.1	Ae. albopictus	Pakistan
MT541498.1	Ae. albopictus	China
MT541368.1	Ae. albopictus	China
MT541282.1	Ae. albopictus	China
MT541271.1	Ae. albopictus	China
MT541270.1	Ae. albopictus	China
KU497592.1	Ae. albopictus	China
KM502237.1	Ae. albopictus	China
KF406542.1	Ae. albopictus	Pakistan
MN540322.1	Ae. albopictus	Malaysia
MN509207.1	Ae. albopictus	USA
MT541164.1	Ae. albopictus	China
HM102286.1	Ae. albopictus	USA
KU497590.1	Ae. albopictus	China
KM502240.1	Ae. albopictus	China
MZ828134.1	Ae. albopictus	India
MZ828133.1	Ae. albopictus	India
MT541170.1	Ae. albopictus	China

KY817527.1	Ae. albopictus	Malaysia
KF406508.1	Ae. albopictus	Pakistan
KF406470.1	Ae. albopictus	Pakistan
MW577641.1	Ae. albopictus	Algeria
KU497591.1	Ae. albopictus	China
MF059293.1	Ae. albopictus	Malta
KY817560.1	Ae. albopictus	Malaysia
KM502244.1	Ae. albopictus	China
MT278262.1	Ae. albopictus	Algeria
MN064773.1	Ae. albopictus	Palestine
MN064772.1	Ae. albopictus	Palestine
MN064771.1	Ae. albopictus	Palestine
MN064770.1	Ae. albopictus	Palestine
MN064769.1	Ae. albopictus	Palestine
MN064768.1	Ae. albopictus	Palestine
MN064767.1	Ae. albopictus	Palestine
MN064766.1	Ae. albopictus	Palestine
MN064765.1	Ae. albopictus	Palestine
MN064764.1	Ae. albopictus	Palestine
MN064763.1	Ae. albopictus	Palestine
MN064762.1	Ae. albopictus	Palestine
MN064761.1	Ae. albopictus	Palestine
MN064760.1	Ae. albopictus	Palestine
MN064759.1	Ae. albopictus	Palestine
MN064757.1	Ae. albopictus	Palestine
MN064756.1	Ae. albopictus	Palestine
MN064755.1	Ae. albopictus	Palestine
MN064754.1	Ae. albopictus	Palestine
KF406554.1	Ae. albopictus	Pakistan
KX266676.1	Ae. albopictus	China
KF406424.1	Ae. albopictus	Pakistan
KF406409.1	Ae. albopictus	Pakistan
KX495910.1	Ae. albopictus	Viet Nam
KF406521.1	Ae. albopictus	Pakistan
KF406516.1	Ae. albopictus	Pakistan
MK518354.1	Ae. albopictus	Serbia
KF135494.1	Ae. albopictus	Mayotte
KX495925.1	Ae. albopictus	Viet Nam
KX495923.1	Ae. albopictus	Viet Nam
KX495915.1	Ae. albopictus	Viet Nam
KX495911.1	Ae. albopictus	Viet Nam
KX573912.1	Ae. albopictus	Viet Nam
JN406797.1	Ae. albopictus	Madagascar

JN406666.1	Ae. albopictus	Madagascar
JN406654.1	Ae. albopictus	Reunion
DQ310142.1	Ae. albopictus	India
MN064753.1	Ae. albopictus	Palestine
KY817537.1	Ae. albopictus	Malaysia
KX573911.1	Ae. albopictus	Viet Nam
KX495924.1	Ae. albopictus	Viet Nam
JN406805.1	Ae. albopictus	Madagascar
JN406796.1	Ae. albopictus	Madagascar
JN406691.1	Ae. albopictus	Madagascar
JN406664.1	Ae. albopictus	Madagascar
JN406659.1	Ae. albopictus	Reunion
MN064758.1	Ae. albopictus	Palestine
MF101840.1	Ae. albopictus	ecuador
KY817555.1	Ae. albopictus	Malaysia
KF406464.1	Ae. albopictus	Pakistan
JN406729.1	Ae. albopictus	Madagascar
JN406795.1	Ae. albopictus	Madagascar
JN406675.1	Ae. albopictus	Madagascar
MK377015.1	Ae. albopictus	India
KY817556.1	Ae. albopictus	Malaysia
KY817554.1	Ae. albopictus	Malaysia
KF406408.1	Ae. albopictus	Pakistan
JN406661.1	Ae. albopictus	Reunion
KY817544.1	Ae. albopictus	Malaysia
KY817564.1	Ae. albopictus	Malaysia
KP211400.1	Ae. albopictus	brazil
MG198596.1	Ae. albopictus	Russia
JN406657.1	Ae. albopictus	Reunion
KY378915.1	Ae. albopictus	China
JN406658.1	Ae. albopictus	Reunion
JN406655.1	Ae. albopictus	Reunion
KY378938.1	Ae. albopictus	China
KY378914.1	Ae. albopictus	China
JN406656.1	Ae. albopictus	Reunion
KY378937.1	Ae. albopictus	China
KY378934.1	Ae. albopictus	China
KY378933.1	Ae. albopictus	China
KY378931.1	Ae. albopictus	China
KY378930.1	Ae. albopictus	China
KY378929.1	Ae. albopictus	China
KY378928.1	Ae. albopictus	China
KY378927.1	Ae. albopictus	China

KY378926.1	Ae. albopictus	China
KY378923.1	Ae. albopictus	China
KY378921.1	Ae. albopictus	China
KY378916.1	Ae. albopictus	China
KY378936.1	Ae. albopictus	China
KY378932.1	Ae. albopictus	China
KY378925.1	Ae. albopictus	China
KY378924.1	Ae. albopictus	China
KY378922.1	Ae. albopictus	China
KY378919.1	Ae. albopictus	China
KY817546.1	Ae. albopictus	Malaysia
KY378920.1	Ae. albopictus	China
KY378917.1	Ae. albopictus	China
KP896571.1	Ae. albopictus	Malaysia
KY817557.1	Ae. albopictus	Malaysia
KY817540.1	Ae. albopictus	Malaysia
KY817539.1	Ae. albopictus	Malaysia
KY817561.1	Ae. albopictus	Malaysia
KY817532.1	Ae. albopictus	Malaysia
KF406536.1	Ae. albopictus	Pakistan
KY817562.1	Ae. albopictus	Malaysia
KX227736.1	Ae. albopictus	India
KY817566.1	Ae. albopictus	Malaysia
KP896554.1	Ae. albopictus	Malaysia
KF406413.1	Ae. albopictus	Pakistan
KY817531.1	Ae. albopictus	Malaysia
KY817528.1	Ae. albopictus	Malaysia
KX227738.1	Ae. albopictus	India
KF406443.1	Ae. albopictus	Pakistan
MN540323.1	Ae. albopictus	Malaysia
KY817547.1	Ae. albopictus	Malaysia
KP896560.1	Ae. albopictus	Malaysia
KY817538.1	Ae. albopictus	Malaysia
KF406515.1	Ae. albopictus	Pakistan
MW270081.1	Ae. albopictus	Indonesia
MW270078.1	Ae. albopictus	Indonesia
AJ971003.1	Ae. albopictus	brazil
AJ971008.1	Ae. albopictus	France
KP896566.1	Ae. albopictus	Malaysia
OK637336.1	Ae. albopictus	USA
MW843029.1	Ae. albopictus	France
MW843028.1	Ae. albopictus	France
MW270084.1	Ae. albopictus	Indonesia

AJ971010.1	Ae. albopictus	Viet Nam
KP896564.1	Ae. albopictus	Malaysia
AJ971014.1	Ae. albopictus	brazil
AJ971005.1	Ae. albopictus	USA
KY817551.1	Ae. albopictus	Malaysia
KY817559.1	Ae. albopictus	Malaysia
KF406479.1	Ae. albopictus	Pakistan
AJ971006.1	Ae. albopictus	Cambodia
KP896568.1	Ae. albopictus	Malaysia
AJ971009.1	Ae. albopictus	France
AJ971007.1	Ae. albopictus	Madagascar
MW270139.1	Ae. albopictus	Indonesia
MW270133.1	Ae. albopictus	Indonesia
KY817523.1	Ae. albopictus	Malaysia
KF406514.1	Ae. albopictus	Pakistan
AJ971015.1	Ae. albopictus	Thailand
AJ971012.1	Ae. albopictus	Reunion
MW270145.1	Ae. albopictus	Indonesia
AJ971011.1	Ae. albopictus	USA
OK637343.1	Ae. albopictus	USA
KY817542.1	Ae. albopictus	Malaysia
KY817543.1	Ae. albopictus	Malaysia
AJ971013.1	Ae. albopictus	Reunion
KY817541.1	Ae. albopictus	Malaysia
KY817529.1	Ae. albopictus	Malaysia
MK182261.1	Ae. albopictus	Pakistan
KY817550.1	Ae. albopictus	Malaysia
KF657725.1	Ae. albopictus	Belgium
MT345349.1	Ae. albopictus	Democratic Republic of the Congo
KY817545.1	Ae. albopictus	Malaysia
JX649856.1	Ae. albopictus	India
MT345390.1	Ae. albopictus	Democratic Republic of the Congo
HF536717.1	Ae. albopictus	Romania
KT260198.1	Ae. albopictus	India
OK637342.1	Ae. albopictus	USA
KT260197.1	Ae. albopictus	India
KP334275.1	Ae. albopictus	Indonesia
KT260199.1	Ae. albopictus	India
KY817548.1	Ae. albopictus	Malaysia
KP334274.1	Ae. albopictus	Indonesia
JX675570.1	Ae. albopictus	germany
AY729984.1	Ae. albopictus	India
MT651311.1	Ae. albopictus	China

MN909291.1	Ae. albopictus	Pakistan
MT651320.1	Ae. albopictus	China
MT651319.1	Ae. albopictus	China
MT651310.1	Ae. albopictus	China
MT651309.1	Ae. albopictus	China
MT651301.1	Ae. albopictus	China
KF406441.1	Ae. albopictus	Pakistan
MT651325.1	Ae. albopictus	China
MT651324.1	Ae. albopictus	China
MT651323.1	Ae. albopictus	China
MT651322.1	Ae. albopictus	China
MT651321.1	Ae. albopictus	China
MT651318.1	Ae. albopictus	China
MT651317.1	Ae. albopictus	China
MT651316.1	Ae. albopictus	China
MT651315.1	Ae. albopictus	China
MT651314.1	Ae. albopictus	China
MT651313.1	Ae. albopictus	China
MT651312.1	Ae. albopictus	China
MT651308.1	Ae. albopictus	China
MT651304.1	Ae. albopictus	China
MT651302.1	Ae. albopictus	China
MG756656.1	Ae. albopictus	India
MT651307.1	Ae. albopictus	China
MT651306.1	Ae. albopictus	China
MT651305.1	Ae. albopictus	China
MT651303.1	Ae. albopictus	China
KY765459.1	Ae. albopictus	China
KF406459.1	Ae. albopictus	Pakistan
JX456417.1	Ae. albopictus	brazil
KF406416.1	Ae. albopictus	Pakistan
MG756657.1	Ae. albopictus	India
KY817533.1	Ae. albopictus	Malaysia
KM457497.1	Ae. albopictus	The Netherlands
HQ398902.1	Ae. albopictus	Viet Nam
KX886303.1	Ae. albopictus	China
LN808745.1	Ae. albopictus	Romania
AY748238.1	Ae. albopictus	Greece
KM457507.1	Ae. albopictus	The Netherlands
KM457506.1	Ae. albopictus	The Netherlands
KM457503.1	Ae. albopictus	The Netherlands
KM457494.1	Ae. albopictus	The Netherlands
KM457493.1	Ae. albopictus	The Netherlands

KY765466.1	Ae. albopictus	China
KX886308.1	Ae. albopictus	China
KU351080.1	Ae. albopictus	Iran
AY748239.1	Ae. albopictus	Greece
KY817530.1	Ae. albopictus	Malaysia
KX886302.1	Ae. albopictus	China
KX886301.1	Ae. albopictus	China
KX886300.1	Ae. albopictus	China
KX886299.1	Ae. albopictus	China
KX966018.1	Ae. albopictus	algeria
HF912379.1	Ae. albopictus	Albania
HQ906849.1	Ae. albopictus	Croatia
AY834241.1	Ae. albopictus	India
KM457498.1	Ae. albopictus	The Netherlands
KY817526.1	Ae. albopictus	Malaysia
KM457499.1	Ae. albopictus	The Netherlands
KY765464.1	Ae. albopictus	China
KU351081.1	Ae. albopictus	Iran
HQ906848.1	Ae. albopictus	Croatia
MG770598.1	Ae. albopictus	India
MG791857.1	Ae. albopictus	India
MG770601.1	Ae. albopictus	India
MT345360.1	Ae. albopictus	Democratic Republic of the Congo
HQ906850.1	Ae. albopictus	Croatia
KY817534.1	Ae. albopictus	Malaysia
GU299770.1	Ae. albopictus	India
MT345389.1	Ae. albopictus	Democratic Republic of the Congo
HE820720.1	Ae. albopictus	Russia
HQ622905.1	Ae. albopictus	Reunion
KC572173.1	Ae. albopictus	Australia
KY765465.1	Ae. albopictus	China
MG770599.1	Ae. albopictus	India
HQ622907.1	Ae. albopictus	Reunion
MG736660.1	Ae. albopictus	India
KY907293.1	Ae. albopictus	Christmas Island
KC572441.1	Ae. albopictus	Papua New Guinea
KC572352.1	Ae. albopictus	Papua New Guinea
KC572329.1	Ae. albopictus	Papua New Guinea
KC572147.1	Ae. albopictus	Australia
MW193898.1	Ae. albopictus	Hungary
MG198603.1	Ae. albopictus	Russia
HQ622904.1	Ae. albopictus	Seychelles
KY907378.1	Ae. albopictus	Solomon Islands

KY907377.1	Ae. albopictus	Malaysia
KY907376.1	Ae. albopictus	Malaysia
KY907373.1	Ae. albopictus	Malaysia
KY907368.1	Ae. albopictus	Malaysia
KY907367.1	Ae. albopictus	Singapore
KY907366.1	Ae. albopictus	Fiji
KY907337.1	Ae. albopictus	Malaysia
KY907291.1	Ae. albopictus	Myanmar
KC572433.1	Ae. albopictus	Papua New Guinea
KC572192.1	Ae. albopictus	Australia
KC572152.1	Ae. albopictus	Australia
KM502236.1	Ae. albopictus	China
HQ622969.1	Ae. albopictus	madagacar
HQ622945.1	Ae. albopictus	Mayotte
HQ622916.1	Ae. albopictus	Reunion
HQ622939.1	Ae. albopictus	Mayotte
HQ622919.1	Ae. albopictus	Reunion
GU299768.1	Ae. albopictus	India
KY907364.1	Ae. albopictus	Reunion
KY907336.1	Ae. albopictus	Malaysia
KY907292.1	Ae. albopictus	Solomon Islands
KC572330.1	Ae. albopictus	Papua New Guinea
KC572155.1	Ae. albopictus	Australia
MW509605.1	Ae. albopictus	Puerto Rico
KY817549.1	Ae. albopictus	Malaysia
HQ622983.1	Ae. albopictus	Mauritius
HQ622979.1	Ae. albopictus	madagacar
HQ622915.1	Ae. albopictus	Reunion
KY907226.1	Ae. albopictus	Indonesia
KY907224.1	Ae. albopictus	Indonesia
KY907222.1	Ae. albopictus	Australia
KY907221.1	Ae. albopictus	Indonesia
KY907220.1	Ae. albopictus	Indonesia
KY907215.1	Ae. albopictus	Australia
KY907214.1	Ae. albopictus	Malaysia
KY907195.1	Ae. albopictus	Papua New Guinea
KF042881.1	Ae. albopictus	East Timor
KC572184.1	Ae. albopictus	Australia
KC572181.1	Ae. albopictus	Australia
KC572153.1	Ae. albopictus	Australia
KC920752.1	Ae. albopictus	Malaysia
MW283318.1	Ae. albopictus	Indonesia
MW283317.1	Ae. albopictus	Indonesia

MW283314.1	Ae. albopictus	Indonesia
MF284803.1	Ae. albopictus	Slovenia
KU351083.1	Ae. albopictus	iran
KC920788.1	Ae. albopictus	Malaysia
KC920787.1	Ae. albopictus	Malaysia
KC920758.1	Ae. albopictus	Malaysia
KY817552.1	Ae. albopictus	Malaysia
FJ372983.1	Ae. albopictus	India
KY907213.1	Ae. albopictus	Indonesia
KC572218.1	Ae. albopictus	Australia
KC572145.1	Ae. albopictus	Australia
KC920770.1	Ae. albopictus	Malaysia
KC920768.1	Ae. albopictus	Malaysia
KC920767.1	Ae. albopictus	Malaysia
KC920760.1	Ae. albopictus	Malaysia
KC920753.1	Ae. albopictus	Malaysia
KC920751.1	Ae. albopictus	Malaysia
MW283316.1	Ae. albopictus	Indonesia
MW283305.1	Ae. albopictus	Indonesia
KM613121.1	Ae. albopictus	Thailand
KC920786.1	Ae. albopictus	Malaysia
KC920783.1	Ae. albopictus	Malaysia
KC920776.1	Ae. albopictus	Malaysia
KC920762.1	Ae. albopictus	Malaysia
KC920757.1	Ae. albopictus	Malaysia
KC920755.1	Ae. albopictus	Malaysia
MF767287.1	Ae. albopictus	Hungary
KC920777.1	Ae. albopictus	Malaysia
KC920774.1	Ae. albopictus	Malaysia
KC920772.1	Ae. albopictus	Malaysia
KC920756.1	Ae. albopictus	Malaysia
MW283315.1	Ae. albopictus	Indonesia
MW283313.1	Ae. albopictus	Indonesia
MW283309.1	Ae. albopictus	Indonesia
MW283308.1	Ae. albopictus	Indonesia
MW283307.1	Ae. albopictus	Indonesia
MW283306.1	Ae. albopictus	Indonesia
MW283304.1	Ae. albopictus	Indonesia
MW283303.1	Ae. albopictus	Indonesia
KC920782.1	Ae. albopictus	Malaysia
KC920780.1	Ae. albopictus	Malaysia
KC920779.1	Ae. albopictus	Malaysia
KC920778.1	Ae. albopictus	Malaysia

KC920766.1	Ae. albopictus	Malaysia
KC920764.1	Ae. albopictus	Malaysia
JF309319.1	Ae. albopictus	Cameroon
KC920769.1	Ae. albopictus	Malaysia
KC920761.1	Ae. albopictus	Malaysia
KC920754.1	Ae. albopictus	Malaysia
MW283312.1	Ae. albopictus	Indonesia
MW283311.1	Ae. albopictus	Indonesia
MW283310.1	Ae. albopictus	Indonesia
KC920785.1	Ae. albopictus	Malaysia
KC920773.1	Ae. albopictus	Malaysia
KC920763.1	Ae. albopictus	Malaysia
KC920759.1	Ae. albopictus	Malaysia
JF309318.1	Ae. albopictus	Cameroon
KC920784.1	Ae. albopictus	Malaysia
KC920781.1	Ae. albopictus	Malaysia
KC920771.1	Ae. albopictus	Malaysia
KY352247.1	Ae. albopictus	Sri Lanka
KM613085.1	Ae. albopictus	Thailand
KM613083.1	Ae. albopictus	Thailand
KC920775.1	Ae. albopictus	Malaysia
KC920765.1	Ae. albopictus	Malaysia
JF309320.1	Ae. albopictus	Cameroon
JF309317.1	Ae. albopictus	Cameroon
LM999972.1	Ae. albopictus	Viet Nam
KY352246.1	Ae. albopictus	Sri Lanka
KY352245.1	Ae. albopictus	Sri Lanka
KM613084.1	Ae. albopictus	Thailand
KC979142.1	Ae. albopictus	Central African Republic
LM999977.1	Ae. albopictus	Viet Nam
LM999976.1	Ae. albopictus	Viet Nam
LM999975.1	Ae. albopictus	France
LM999974.1	Ae. albopictus	France
LM999973.1	Ae. albopictus	France
MN824032.1	Ae. albopictus	China
KP122847.1	Ae. albopictus	Malaysia
KP896573.1	Ae. albopictus	Malaysia
KP896572.1	Ae. albopictus	Malaysia
KY378918.1	Ae. albopictus	China
AY101848.1	Ae. albopictus	China
AY100668.1	Ae. albopictus	China
MN824028.1	Ae. albopictus	China
KY765493.1	Ae. albopictus	China

KP122901.1	Ae. albopictus	Malaysia
KP122900.1	Ae. albopictus	Malaysia
KP122885.1	Ae. albopictus	Malaysia
KP122883.1	Ae. albopictus	Malaysia
KP122867.1	Ae. albopictus	Malaysia
KP122863.1	Ae. albopictus	Malaysia
KP122860.1	Ae. albopictus	Malaysia
KP122853.1	Ae. albopictus	Malaysia
KP122852.1	Ae. albopictus	Malaysia
KP122846.1	Ae. albopictus	Malaysia
KC979143.1	Ae. albopictus	Central African Republic
KP122859.1	Ae. albopictus	Malaysia
OM131687.1	Ae. albopictus	Japan
AY101850.1	Ae. albopictus	China
AY100666.1	Ae. albopictus	China
MN824033.1	Ae. albopictus	China
MN824031.1	Ae. albopictus	China
MN824030.1	Ae. albopictus	China
MN824029.1	Ae. albopictus	China
KP122905.1	Ae. albopictus	Malaysia
KP122896.1	Ae. albopictus	Malaysia
KP122890.1	Ae. albopictus	Malaysia
KP122889.1	Ae. albopictus	Malaysia
KP122886.1	Ae. albopictus	Malaysia
KP122882.1	Ae. albopictus	Malaysia
KP122875.1	Ae. albopictus	Malaysia
KP122874.1	Ae. albopictus	Malaysia
KP122873.1	Ae. albopictus	Malaysia
KP122872.1	Ae. albopictus	Malaysia
KP122869.1	Ae. albopictus	Malaysia
KP122864.1	Ae. albopictus	Malaysia
KP122861.1	Ae. albopictus	Malaysia
KP122858.1	Ae. albopictus	Malaysia
KP122856.1	Ae. albopictus	Malaysia
KP122855.1	Ae. albopictus	Malaysia
KP122854.1	Ae. albopictus	Malaysia
KP122850.1	Ae. albopictus	Malaysia
KP122849.1	Ae. albopictus	Malaysia
KP122909.1	Ae. albopictus	Malaysia
KP122904.1	Ae. albopictus	Malaysia
KP122881.1	Ae. albopictus	Malaysia
KP122895.1	Ae. albopictus	Malaysia
KF406423.1	Ae. albopictus	Pakistan

AY101849.1	Ae. albopictus	China
AY100667.1	Ae. albopictus	China
MN824039.1	Ae. albopictus	China
MN824037.1	Ae. albopictus	China
MN824036.1	Ae. albopictus	China
MN824035.1	Ae. albopictus	China
MN824034.1	Ae. albopictus	China
MN824027.1	Ae. albopictus	China
KP122893.1	Ae. albopictus	Malaysia
KP122888.1	Ae. albopictus	Malaysia
KP122887.1	Ae. albopictus	Malaysia
KP122880.1	Ae. albopictus	Malaysia
KP122878.1	Ae. albopictus	Malaysia
KP122877.1	Ae. albopictus	Malaysia
KP122871.1	Ae. albopictus	Malaysia
KP122870.1	Ae. albopictus	Malaysia
KP122866.1	Ae. albopictus	Malaysia
KP122865.1	Ae. albopictus	Malaysia
KP122857.1	Ae. albopictus	Malaysia
KP122848.1	Ae. albopictus	Malaysia
KP122879.1	Ae. albopictus	Malaysia
KP122907.1	Ae. albopictus	Malaysia
KP122899.1	Ae. albopictus	Malaysia
KP122898.1	Ae. albopictus	Malaysia
MN824038.1	Ae. albopictus	China
KP122903.1	Ae. albopictus	Malaysia
KP122876.1	Ae. albopictus	Malaysia
KP122868.1	Ae. albopictus	Malaysia
KP122862.1	Ae. albopictus	Malaysia
KP122851.1	Ae. albopictus	Malaysia
JQ436969.1	Ae. albopictus	Viet Nam
JQ436978.1	Ae. albopictus	Thailand
AJ971004.1	Ae. albopictus	Viet Nam
MH348258.1	Ae. albopictus	Balearic Islands
KP122906.1	Ae. albopictus	Malaysia
KP122902.1	Ae. albopictus	Malaysia
JQ436980.1	Ae. albopictus	Thailand
JQ436979.1	Ae. albopictus	Thailand
JQ436977.1	Ae. albopictus	Thailand
JQ436974.1	Ae. albopictus	Bhutan
JQ436972.1	Ae. albopictus	Viet Nam
JQ436970.1	Ae. albopictus	Viet Nam
JQ436960.1	Ae. albopictus	Japan

MT434294.1	Ae. albopictus	Viet Nam
KP122892.1	Ae. albopictus	Malaysia
KP122884.1	Ae. albopictus	Malaysia
OK465301.1	Ae. albopictus	China
OK465300.1	Ae. albopictus	China
OK465271.1	Ae. albopictus	China
OK465270.1	Ae. albopictus	China
JQ436975.1	Ae. albopictus	Bhutan
JQ436973.1	Ae. albopictus	Viet Nam
JQ436971.1	Ae. albopictus	Viet Nam
JQ436966.1	Ae. albopictus	China
JQ436965.1	Ae. albopictus	China
JQ436964.1	Ae. albopictus	China
JQ436961.1	Ae. albopictus	Japan
KP122891.1	Ae. albopictus	Malaysia
JQ436976.1	Ae. albopictus	Bhutan
JQ436968.1	Ae. albopictus	China
JQ436967.1	Ae. albopictus	China
JQ436963.1	Ae. albopictus	China
JQ436962.1	Ae. albopictus	Japan
KP122908.1	Ae. albopictus	Malaysia
OK465293.1	Ae. albopictus	China
OK465292.1	Ae. albopictus	China
AY101847.1	Ae. albopictus	China
KY378935.1	Ae. albopictus	China
LT221029.1	Ae. albopictus	Greece
KP122897.1	Ae. albopictus	Malaysia
KP122894.1	Ae. albopictus	Malaysia
OK465269.1	Ae. albopictus	China
GU299769.1	Ae. albopictus	India
KF406401.1	Ae. albopictus	Pakistan
MW159865.1	Ae. albopictus	colombia
MW159880.1	Ae. albopictus	colombia
MW159866.1	Ae. albopictus	colombia
KF406418.1	Ae. albopictus	Pakistan
MW159900.1	Ae. albopictus	colombia
MW159899.1	Ae. albopictus	colombia
MW159884.1	Ae. albopictus	colombia
MW159864.1	Ae. albopictus	colombia
MW159901.1	Ae. albopictus	colombia
MW159881.1	Ae. albopictus	colombia
KF406461.1	Ae. albopictus	Pakistan
KF406548.1	Ae. albopictus	Pakistan

MN824041.1	Ae. albopictus	China
MN909290.1	Ae. albopictus	Pakistan
KF406444.1	Ae. albopictus	Pakistan
MN909288.1	Ae. albopictus	Pakistan
MN824040.1	Ae. albopictus	China
KJ765612.1	Ae. albopictus	Malaysia
KJ765611.1	Ae. albopictus	Malaysia
MN909289.1	Ae. albopictus	Pakistan
MG515539.1	Ae. albopictus	China
XR 003899037.1	Ae. albopictus	Italy
XR 003895456.1	Ae. albopictus	Italy
XR 003895455.1	Ae. albopictus	Italy
